# Machine Learning for Graduation Prediction in Higher Education: A Systematic Review with a Bio-Inspired Optimization Perspective

**DOI:** 10.3390/biomimetics11070512

**Published:** 2026-07-21

**Authors:** Andrés Yáñez, Broderick Crawford, Eric Monfroy, Álex Paz, José Barrera-García, Felipe Cisternas-Caneo, Álvaro Peña Fritz, Ricardo Soto

**Affiliations:** 1 Escuela de Ingeniería en Construcción y Transporte, Pontificia Universidad Católica de Valparaíso, Avenida Brasil 2147, Valparaíso 2362804, Chile; andres.yanez@pucv.cl (A.Y.); alex.paz@pucv.cl (Á.P.); alvaro.pena@ucv.cl (Á.P.F.); 2 Laboratoire d’Étude et de Recherche en Informatique d’Angers (LERIA), Université d’Angers, 49000 Angers, France; eric.monfroy@univ-angers.fr; 3 Escuela de Ingeniería Informática, Pontificia Universidad Católica de Valparaíso, Avenida Brasil 2241, Valparaíso 2362807, Chile; felipe.cisternas.c@mail.pucv.cl (F.C.-C.); ricardo.soto@pucv.cl (R.S.); 4 Escuela de Negocios y Economía, Pontificia Universidad Católica de Valparaíso, Amunátegui 1838, Viña del Mar 2580129, Chile; 5 Department of Computer Science, Universidad de Alcalá, 28805 Madrid, Spain

**Keywords:** graduation prediction, higher education, machine learning, systematic literature review, educational analytics, bio-inspired optimization, explainable artificial intelligence

## Abstract

Timely graduation, time-to-degree, and degree completion are key indicators of student progression and institutional effectiveness in higher education. This study presents a PRISMA-based systematic literature review of machine learning approaches for graduation-related prediction, with attention to predictive targets, pipeline components, scalability, and bio-inspired optimization. Searches in Web of Science Core Collection and Scopus identified 278 records, of which 25 studies published between 2021 and 2025 met the eligibility criteria. The findings show that most studies formulated graduation prediction as a supervised classification task, relied heavily on academic performance variables, and frequently used tree-based or ensemble models. Feature selection, explainability, and hyperparameter optimization were commonly reported, but bio-inspired optimization was actively implemented in only two studies through Particle Swarm Optimization, Genetic Algorithms, or Ant Colony Optimization. The evidence base also remains limited in scalability, as most studies used single-institution datasets and provided little external validation. These findings identify an opportunity for Bio-Inspired Educational Analytics through scalable feature selection, efficient hyperparameter optimization, model simplification, and multi-objective trade-off analysis. Future research should evaluate whether lightweight, hybrid, and multi-objective metaheuristics can support accurate, interpretable, fair, and transferable graduation prediction systems.

## 1. Introduction

Timely graduation, time-to-degree (TTD), and degree completion are central concerns for higher education institutions because they reflect not only student progression but also institutional capacity, resource allocation, and educational equity. Delays in degree completion can increase financial pressure on students and families, extend opportunity costs, and affect institutional planning by limiting the capacity to admit and support new cohorts [1,2]. These effects are not distributed evenly across student populations. Students from vulnerable, rural, or historically underrepresented groups may face additional academic, financial, and social barriers that increase the risk of delayed completion or non-completion [3,4]. For this reason, predicting graduation-related outcomes has become increasingly relevant for institutions seeking to design earlier and more targeted support strategies.

In this context, data-driven approaches have gained attention as tools for identifying students who may require academic or institutional support. Learning analytics and predictive learning analytics have been widely used to support study success in higher education, particularly through early identification of students at risk and the use of institutional, academic, and digital data for decision support [5,6]. Machine learning, statistical learning, and educational data mining methods have also been applied to predict on-time graduation, degree completion, student completion, delayed graduation, and time-to-degree using institutional, academic, sociodemographic, behavioral, financial, and curricular data [7,8,9]. These approaches are attractive because they can model nonlinear relationships and interactions among variables that are difficult to capture through traditional descriptive monitoring.

However, the use of predictive models in higher education raises methodological and practical challenges. First, graduation-related outcomes are often operationalized differently across studies, including on-time graduation, degree completion, TTD, delayed graduation, and completion-related risk. These outcomes may require different modeling strategies, validation procedures, and evaluation metrics. Second, the predictors used in these models vary substantially, ranging from academic performance and admission records to socioeconomic, institutional, curricular, behavioral, and longitudinal variables. Third, models developed from institutional data must be interpreted carefully because strong predictive variables may reflect existing structural inequalities rather than directly actionable mechanisms. This concern is consistent with broader evidence on predictive models in education, which highlights risks related to bias, transparency, interpretability, data processing heterogeneity, and limited generalizability across institutional contexts [10,11]. Therefore, predictive models for graduation-related outcomes should be evaluated not only in terms of aggregate performance, but also in terms of subgroup behavior, fairness, transparency, and the educational consequences of their use.

Explainability has become particularly important in this field because higher education decision-makers need to understand why a model classifies a student as being at risk of delayed graduation or non-completion. Methods such as SHAP and LIME have been incorporated in some studies to provide global and local explanations of model behavior [7,9,12]. Nevertheless, explainability is still unevenly integrated across the literature. Some studies rely on global feature importance, decision rules, or regression coefficients, while fewer provide case-level explanations that can directly support individualized advising. Recent work on explainable learning analytics further suggests that explanations should not only make model outputs more interpretable, but also support the assessment of model stability across contexts and cohorts [13]. This view aligns with human-centered learning analytics and AI in education, which emphasize stakeholder involvement, trust, transparency, human control, and the conditions under which analytics are translated into educational action [14].

From a biomimetics perspective, graduation-related prediction can be understood as an educational analytics problem involving search, adaptation, and optimization under uncertainty. Institutional datasets often include heterogeneous, redundant, and context-dependent variables, while predictive pipelines must balance several competing requirements, including predictive performance, model parsimony, interpretability, fairness, and computational feasibility. Bio-inspired computation provides a relevant methodological lens for this type of problem because algorithms inspired by biological and collective behaviors, such as evolutionary search, swarm intelligence, and ant-based optimization, have been widely used in computational intelligence to explore large solution spaces, select informative features, and tune model configurations [15,16,17,18]. In the context of graduation prediction, these methods may contribute to feature subset selection, hyperparameter optimization, model simplification, and the analysis of trade-offs between accuracy, interpretability, and responsible institutional use. This perspective is also aligned with the scope of bio-inspired algorithms and scalable systems, because graduation prediction requires not only accurate models, but also predictive pipelines that remain computationally feasible, interpretable, transferable, and adaptable across educational contexts.

Nevertheless, the role of bio-inspired optimization in graduation-related prediction remains insufficiently developed. Although feature selection, dimensionality reduction, and hyperparameter tuning are increasingly reported in educational prediction studies, the use of metaheuristics remains limited in the specific literature on timely graduation, time-to-degree, and degree completion prediction. Existing studies show only isolated applications of Particle Swarm Optimization for hyperparameter tuning and Genetic Algorithms or Ant Colony Optimization for feature selection [19,20]. This limited adoption suggests that the potential contribution of bio-inspired algorithms to educational analytics has not yet been systematically examined, particularly in relation to feature reduction, model tuning, multi-objective optimization, explainability, fairness, and institutional deployment.

Despite the growing number of empirical studies, the literature still lacks a focused synthesis of how machine learning and predictive modeling approaches have been applied specifically to timely graduation, time-to-degree, and degree completion prediction in higher education. Prior reviews have examined broader areas such as predictive learning analytics, student academic success, student performance prediction, or graduation prediction using academic performance data [6,11,21,22]. However, these reviews do not specifically synthesize graduation-related prediction as a distinct completion-oriented problem while simultaneously examining feature selection, dimensionality reduction, explainability, hyperparameter optimization, methodological quality, and bio-inspired optimization. This gap is relevant because recent literature on predictive models in education has emphasized not only predictive performance, but also transparency, interpretability, generalizability, and responsible use in institutional decision-making [11,13].

This distinction is also important because adjacent outcomes such as dropout, retention, persistence, academic achievement, course performance, and first-year progression are conceptually related to student success, but they do not necessarily represent the same prediction target. Dropout prediction usually focuses on identifying students likely to leave before completion, retention prediction often concerns continued enrollment between academic periods, persistence refers to sustained progression through a program, and academic achievement typically captures grades or performance indicators [6,21]. By contrast, graduation-related prediction focuses on whether, when, or within what expected period students complete a degree [22,23]. Therefore, studies centered exclusively on dropout, retention, persistence, or academic achievement were considered adjacent literature and were excluded unless they explicitly modeled graduation, degree completion, student completion, or time-to-degree. A dedicated systematic literature review is therefore needed to clarify which modeling approaches have been used, which predictors are commonly reported as relevant, how feature selection, explainability, hyperparameter optimization, and bio-inspired optimization are incorporated into predictive pipelines, and which methodological gaps remain for developing future Bio-Inspired Educational Analytics [11,22].

Accordingly, this study presents a systematic literature review of machine learning and predictive modeling approaches for timely graduation, time-to-degree, and degree completion prediction in higher education. The novelty of this review lies in its focused treatment of graduation-related prediction as a distinct educational analytics problem, rather than as a broad extension of dropout, retention, or academic performance prediction [6,21,22]. This distinction allows the review to synthesize evidence specifically related to whether, when, or within what expected period students complete a degree, which has been addressed in prior empirical studies through outcomes such as degree completion, time-to-degree, delayed graduation, and on-time graduation [9,12,23].

The review makes five contributions:

1. First, it provides a PRISMA-based synthesis of recent peer-reviewed studies indexed in WoSCC and Scopus between 2021 and 2025.
2. Second, it characterizes the main prediction targets, modeling approaches, datasets, institutional contexts, evaluation metrics, and predictor categories used in graduation-related prediction.
3. Third, it examines the methodological maturity of predictive pipelines by analyzing feature selection, dimensionality reduction, hyperparameter optimization, explainability, validation strategies, class imbalance treatment, and reporting practices.
4. Fourth, it identifies the limited implementation of bio-inspired optimization and metaheuristic methods as a specific methodological gap within the reviewed corpus.
5. Fifth, it outlines future opportunities for scalable Bio-Inspired Educational Analytics, particularly through feature subset selection, efficient hyperparameter tuning, multi-objective optimization, model simplification, and responsible deployment under institutional constraints.

By connecting graduation-related prediction with bio-inspired optimization, this review contributes to the Biomimetics Special Issue from a methodological perspective. The biomimetic component does not reside in the educational phenomenon itself, but in the computational strategies that may support adaptive search, exploration–exploitation balance, cooperation, selection, and optimization within predictive modeling pipelines. This positioning avoids overstating the empirical evidence in the reviewed corpus while clarifying why bio-inspired computation is relevant for future research on scalable, interpretable, and operationally useful graduation prediction systems. In this sense, the review links the current evidence base with the Special Issue theme by identifying how nature-inspired optimization can support the transition from local predictive models toward scalable educational analytics solutions.

The remainder of the article is organized as follows. Section 2 describes the review design, information sources, search strategy, eligibility criteria, screening process, data extraction framework, methodological quality assessment, and synthesis strategy. Section 3 presents the study selection process, descriptive overview of the included studies, answers to the four research questions, and the methodological quality assessment. Section 4 discusses the main findings, methodological implications, pipeline maturity, responsible deployment issues, and directions for future research. Finally, Section 5 summarizes the conclusions and limitations of the review.

## 2. Methodology

### 2.1. Review Design and Protocol

This study was designed as a systematic literature review of machine learning and predictive modeling approaches for timely graduation, time-to-degree, and degree completion prediction in higher education. The review focused on studies that developed, evaluated, compared, or applied predictive models using student-level, institutional, academic, sociodemographic, behavioral, or related educational data.

The review was conducted and reported in accordance with PRISMA 2020 guidelines [24], to the extent applicable to a narrative synthesis of heterogeneous predictive modeling studies. The review protocol was retrospectively registered in the Open Science Framework (OSF) Registries (https://osf.io/4y82n, accessed on 19 July 2026) during the peer-review process. Before screening and data extraction, a review protocol was defined to guide the search strategy, eligibility criteria, screening decisions, data extraction fields, methodological quality assessment, and evidence synthesis. The protocol was not publicly registered and is not available in an external repository. No amendments were made to the predefined eligibility criteria, search strategy, or synthesis plan after screening began.

The scope of the review included predictive modeling studies addressing timely graduation, on-time graduation, time-to-degree, degree completion, student completion, or equivalent completion outcomes in higher education. Studies were considered eligible when they explicitly framed the task as prediction, classification, forecasting, risk estimation, or decision support. Studies using statistical models were not excluded because of the modeling technique itself; rather, eligibility depended on whether the model was used for a predictive purpose related to student completion.

Given the specific interest of this review in feature selection, explainability, and optimization, the extraction and synthesis also examined whether studies incorporated dimensionality reduction, hyperparameter optimization, explainable artificial intelligence, or bio-inspired optimization methods. The absence of bio-inspired optimization or metaheuristic algorithms was not used as an exclusion criterion. Instead, it was treated as an analytical coding dimension to assess the extent to which these methods have been adopted in the reviewed literature.

### 2.2. Review Objective and Research Questions

The objective of this review was to systematically analyze how machine learning and predictive modeling approaches have been used to predict timely graduation, time-to-degree, and degree completion in higher education. Beyond synthesizing the main modeling approaches, predictor categories, and methodological practices reported in the literature, the review also aimed to identify gaps and future opportunities for incorporating bio-inspired optimization into educational analytics pipelines. Particular attention was given to the role of feature selection, dimensionality reduction, hyperparameter optimization, explainable artificial intelligence, and metaheuristic-based methods as potential components of a future Bio-Inspired Educational Analytics research agenda.

The review was guided by the following research questions:

- **RQ1.** What machine learning and predictive modeling approaches have been used to predict timely graduation, time-to-degree, or degree completion in higher education?
- **RQ2.** What types of predictors have been used and reported as relevant in these prediction models?
- **RQ3.** How have feature selection, dimensionality reduction, hyperparameter optimization, explainable artificial intelligence, and bio-inspired optimization methods been incorporated into timely graduation prediction pipelines?
- **RQ4.** What methodological gaps and future research opportunities emerge for developing Bio-Inspired Educational Analytics in graduation prediction, particularly regarding metaheuristic-based feature selection, hyperparameter optimization, multi-objective modeling, interpretability, fairness, computational cost, and institutional deployment?

### 2.3. Information Sources

The literature search was conducted in the Web of Science Core Collection (WoSCC) and Scopus. These databases were selected because they provide broad multidisciplinary coverage of peer-reviewed research in higher education, educational data mining, computer science, engineering, data science, and applied machine learning.

The search was conducted in January 2026. The review was restricted to peer-reviewed articles written in English and published between 2021 and 2025. Only documents classified as articles were considered eligible. This restriction was adopted to retain full-length studies with sufficient methodological detail for screening, data extraction, methodological quality assessment, and evidence synthesis.

No additional database-specific restrictions were imposed beyond publication year, document type, language, and the search fields available in each database. Records retrieved from both databases were exported with bibliographic metadata, abstracts, keywords, DOI, source information, and indexing information when available.

### 2.4. Search Strategy

The search strategy combined three conceptual blocks: graduation-related outcomes, predictive modeling terms, and higher education context terms. Graduation-related terms were used to retrieve studies addressing timely graduation, on-time graduation, time-to-degree, degree completion, student completion, and equivalent completion outcomes. Predictive modeling terms were included to focus the search on studies using models for prediction or forecasting. Higher education terms were included to restrict the search to university, college, undergraduate, tertiary education, or comparable postsecondary contexts.

The same conceptual search strategy was adapted to the syntax and search fields of each database. In WoSCC, the search was conducted using the Topic field. The search query was:

(“timely graduation” OR “on-time graduation” OR “ontime graduation” OR “on time graduation” OR “time to degree” OR “time-to-degree” OR “degree completion” OR “student completion” OR “ontime completion” OR “on time completion” OR “on-time completion” OR “On-time Student Graduation”) Topic AND (model OR modeling OR prediction OR predictive) Topic AND (“higher education” OR university OR college OR undergraduate OR “tertiary education” OR major) Topic AND English Languages AND Article Document Types AND (2025 OR 2024 OR 2023 OR 2022 OR 2021) Final Publication Years

In Scopus, the search was conducted using the TITLE-ABS-KEY field. The search query was:

(TITLE-ABS-KEY(“timely graduation” OR “on-time graduation” OR “ontime graduation” OR “on time graduation” OR “time to degree” OR “time-to-degree” OR “degree completion” OR “student completion” OR “ontime completion” OR “on time completion” OR “on-time completion” OR “On-time Student Graduation”) AND TITLE-ABS-KEY(model OR modeling OR prediction OR predictive) AND TITLE-ABS-KEY(“higher education” OR university OR college OR undergraduate OR “tertiary education” OR major)) AND (LIMIT-TO (DOCTYPE, “ar”)) AND (LIMIT-TO (LANGUAGE, “English”)) AND PUBYEAR > 2020 AND PUBYEAR < 2026

The search strings, database filters, and retrieval dates were recorded to support reproducibility.

### 2.5. Record Management and Duplicate Removal

Records retrieved from WoSCC and Scopus were exported and consolidated into a single screening dataset. Duplicate records were identified using DOI as the primary matching criterion. When DOI information was missing, incomplete, or inconsistent, records were compared using title, authors, publication year, and source title.

When duplicate records were found across databases, a single record was retained for screening. Preference was given to the record with the most complete bibliographic metadata, including abstract, keywords, DOI, and source information. The deduplicated dataset was then used for title and abstract screening.

The number of records identified in each database, duplicate records removed, records screened, records excluded, reports assessed for full-text eligibility, and studies included in the final synthesis was documented in the PRISMA flow diagram.

### 2.6. Eligibility Criteria

Eligibility criteria were defined before the screening process and were applied consistently during title and abstract screening and full-text assessment. A study was included only when it satisfied all inclusion criteria. Records were excluded when at least one exclusion criterion applied.

#### 2.6.1. Inclusion Criteria

- **IC1. Eligible educational context.** The study had to be situated in higher education, university, college, undergraduate education, tertiary education, or an equivalent postsecondary context.
- **IC2. Eligible outcome of interest.** The study had to address timely graduation, on-time graduation, time-to-degree, degree completion, student completion, completion within an expected period, or a directly equivalent outcome.
- **IC3. Eligible predictive approach.** The study had to use, evaluate, propose, or compare a predictive model, machine learning method, artificial intelligence approach, data mining technique, statistical learning method, or a comparable computational approach.
- **IC4. Relevant data for prediction.** The study had to use student-level, institutional, academic, demographic, behavioral, curricular, socioeconomic, or other predictors related to degree completion.
- **IC5. Relevance to the research questions.** The study had to provide evidence on at least one of the following dimensions: predictive models, predictors, feature selection, dimensionality reduction, explainability, hyperparameter optimization, bio-inspired optimization, metaheuristics, validation, reproducibility, or methodological gaps in graduation or completion prediction.

For clarity, adjacent student-success outcomes were treated as eligible only when they were explicitly linked to a graduation-related prediction target. Thus, studies addressing dropout, retention, persistence, academic achievement, course performance, or first-year progression were included only when they explicitly modeled graduation, degree completion, student completion, completion within an expected period, or time-to-degree. Studies focused on these adjacent outcomes without an explicit graduation-related or completion-related target were excluded to preserve conceptual alignment with the review objective.

#### 2.6.2. Exclusion Criteria

- **EC1. Ineligible educational context.** The study was not situated in higher education, university, college, undergraduate education, tertiary education, or an equivalent postsecondary context.
- **EC2. Ineligible population.** The study focused exclusively on postgraduate education, master’s programs, doctoral programs, medical residency, professional certification, secondary education, primary education, or non-university education, unless it explicitly analyzed undergraduate degree completion.
- **EC3. Ineligible main outcome.** The main outcome did not correspond to timely graduation, on-time graduation, time-to-degree, degree completion, student completion, completion within an expected period, or a directly equivalent outcome.
- **EC4. Adjacent outcome without an explicit link to graduation or completion.** The study focused on dropout, retention, persistence, course performance, first-year success, academic achievement, GPA, employability, well-being, satisfaction, or engagement without explicitly modeling graduation, degree completion, student completion, completion within an expected period, or time-to-degree.
- **EC5. Absence of predictive modeling.** The study did not use predictive modeling, machine learning, artificial intelligence, data mining, statistical learning, or a comparable computational approach.
- **EC6. Non-predictive or non-empirical study.** The study was qualitative, theoretical, conceptual, descriptive, policy-oriented, practice-based, exploratory, or a review article without a predictive modeling objective.
- **EC7. Statistical model with an explanatory, causal, or associative purpose.** The study used regression, structural equation modeling, survival analysis, econometric models, association models, or retrospective analyses only to explain relationships, evaluate effects, validate constructs, or estimate impacts, but not for prediction, classification, forecasting, risk estimation, or decision support.
- **EC8. Outcome limited to a course, subject, exam, or first-year progression.** The study predicted performance in a course, subject, module, exam, GPA-only outcome, course passing, or first-year progression without addressing completion of the full academic program.
- **EC9. False positive outside the educational domain.** The study belonged to another domain, such as construction, logistics, health, engineering projects, or another field unrelated to higher education.
- **EC10. Ineligible documentary criteria.** The record did not meet the documentary criteria defined for the review: source database limited to Web of Science Core Collection or Scopus, English language, article document type, and publication period from 2021 to 2025.

Statistical models were not excluded on the basis of their methodological family. Studies using regression, survival analysis, econometric models, structural equation modeling, or related statistical approaches were eligible when these methods were used for prediction, classification, forecasting, risk estimation, or decision-support purposes and included some form of predictive evaluation. Conversely, studies were excluded under EC7 when the model was used exclusively to explain associations, estimate effects, test hypotheses, validate constructs, or support causal or impact-oriented interpretation without a predictive modeling objective.

The absence of feature selection, explainable artificial intelligence, bio-inspired optimization, or metaheuristic methods was not used as an exclusion criterion. These elements were treated as analytical coding dimensions during data extraction and synthesis.

### 2.7. Study Selection Process

The study selection process was conducted in two sequential stages. First, titles and abstracts were screened against the predefined eligibility criteria. Records were classified as eligible, excluded, borderline, or requiring full-text verification. Records were excluded at this stage only when the title and abstract provided sufficient evidence that they did not meet the review criteria. Borderline or unclear records were retained for full-text assessment to reduce the risk of erroneous exclusion.

The title and abstract screening was conducted independently by two reviewers using the predefined inclusion and exclusion criteria. Disagreements were resolved through discussion and consensus. When eligibility remained uncertain after discussion, the record was retained for full-text assessment in order to minimize the risk of excluding potentially relevant studies.

Second, the full texts of all potentially eligible reports were assessed using the same inclusion and exclusion criteria. At this stage, each report was reviewed to confirm whether it addressed an eligible higher education context, an eligible graduation- or completion-related outcome, and a predictive or computational modeling approach. Reports were excluded when the full text showed that the population, outcome, methodological approach, document type, or educational context did not meet the predefined criteria.

Full-text assessment was also conducted by two reviewers. Disagreements were resolved through discussion within the review team, with final decisions based on the predefined eligibility criteria and the conceptual alignment of each study with the review scope. Particular attention was given to whether the study explicitly modeled timely graduation, time-to-degree, degree completion, student completion, completion within an expected period, or a directly equivalent outcome.

The numerical flow of records through the identification, duplicate removal, title and abstract screening, full-text assessment, and final inclusion stages is reported in the Section 3 using a PRISMA-based summary.

The final corpus reflects the specific scope of the review, which focused on predictive or computational approaches explicitly addressing timely graduation, on-time graduation, time-to-degree, degree completion, student completion, completion within an expected period, or directly equivalent outcomes in higher education.

### 2.8. Data Extraction and Coding Framework

A structured extraction matrix was designed to collect bibliographic, methodological, predictive, and technical information from each included study. The extraction process was organized according to the research questions and was intended to support a consistent comparison of studies with different prediction targets, datasets, modeling approaches, and reporting practices.

Data extraction was conducted by two reviewers using the structured extraction matrix. Each reviewer extracted the relevant information from the included studies, and the extracted data were subsequently cross-checked for consistency. Disagreements, unclear cases, or ambiguities in coding were resolved through discussion within the review team. No study investigators were contacted because all extracted data were obtained from the published reports.

The extracted information included bibliographic details, study context, prediction task, dataset characteristics, predictors, modeling methods, validation strategy, evaluation metrics, feature selection procedures, dimensionality reduction methods, explainability methods, hyperparameter optimization techniques, bio-inspired optimization methods, institutional implications, and reported limitations. When information was not explicitly reported, it was coded as ‘Not reported’. When the information was ambiguous or insufficiently described, it was coded as ‘Unclear’.

To avoid ambiguity during coding, feature selection and dimensionality reduction were treated as distinct methodological categories. Feature selection refers to procedures that identify and retain a subset of the original input variables, such as filter, wrapper, embedded, or metaheuristic-based selection methods [25,26,27]. Dimensionality reduction refers to procedures that transform the original feature space into a lower-dimensional representation, such as principal component analysis, latent representations, embeddings, or other projection-based techniques [28,29,30]. This distinction was used during data extraction to differentiate studies that reduced the number of original predictors from those that transformed predictors into new derived components.

Predictors were grouped into academic performance, sociodemographic, socioeconomic or financial, institutional, behavioral, curricular or program-level, pre-college or admission-related, and longitudinal academic trajectory categories. This categorization was used to compare the types of information most frequently used in graduation-related prediction and to identify whether selected predictors were primarily academic, contextual, behavioral, institutional, or trajectory-based.

Modeling approaches were classified into traditional machine learning models, tree-based ensemble methods, neural networks and deep learning approaches, statistical predictive models, hybrid approaches, and other computational models. In addition, the role of each model within the predictive pipeline was coded when reported, including baseline model, main predictive model, comparative model, ensemble component, or final deployed or recommended model.

Feature selection, dimensionality reduction, hyperparameter optimization, explainability, and bio-inspired optimization were coded as specific pipeline components. Feature selection and dimensionality reduction were coded according to the method used, the stage of the pipeline in which they were applied, and whether they contributed to performance improvement, model simplification, or interpretability. Hyperparameter optimization was coded according to whether it was manual, grid-based, random, automated, Bayesian, metaheuristic-based, or not reported.

For bio-inspired optimization and metaheuristics, each study was coded according to whether these methods were absent, mentioned only in the background, actively implemented, or unclear. When actively implemented, the algorithm name, bio-inspired family, role in the predictive pipeline, optimization target, comparison strategy, and reported effect on model performance, feature reduction, model parsimony, interpretability, or computational efficiency were extracted. The coding also distinguished whether the method was used for feature selection, hyperparameter tuning, model selection, ensemble construction, multi-objective optimization, or another optimization task.

Finally, additional coding dimensions were included to support the identification of future opportunities for Bio-Inspired Educational Analytics. These dimensions considered whether each study addressed interpretability, fairness, class imbalance, external validation, computational cost, institutional deployment, or actionability of model outputs. This allowed the synthesis to examine not only the current use of machine learning techniques, but also the extent to which existing pipelines provide methodological foundations for future bio-inspired, multi-objective, explainable, and responsible educational analytics approaches.

### 2.9. Methodological Quality Assessment Procedure

A methodological quality assessment was conducted to evaluate the transparency, reliability, reproducibility, and potential risk of bias of the included studies. Because the included articles were heterogeneous predictive modeling studies rather than intervention studies, a domain-specific methodological quality assessment was used instead of a conventional clinical risk-of-bias tool. The purpose of this assessment was not to exclude studies from the synthesis, but to contextualize the evidence and identify recurring methodological limitations across the corpus.

Each study was assessed using criteria aligned with predictive modeling research in educational settings. The criteria examined whether the study provided: (i) a clear definition of the prediction target; (ii) an adequate description of the dataset and study population; (iii) transparent reporting of predictors or input features; (iv) a description of data preprocessing procedures; (v) an appropriate training, validation, and testing strategy; (vi) consideration of class imbalance when applicable; (vii) suitable evaluation metrics; (viii) comparison with baseline or alternative models; (ix) reporting of feature selection, explainability, or optimization procedures when applicable; and (x) discussion of limitations, generalizability, and potential bias.

Each criterion was coded as satisfied, partially satisfied, not satisfied, or not reported. The assessment was conducted by two reviewers, and disagreements or unclear cases were resolved through discussion within the review team. The results of the assessment were used to support the interpretation of the findings and to identify areas where future studies could improve reporting, validation, reproducibility, and bias control.

### 2.10. Synthesis Strategy

Given the heterogeneity of prediction targets, datasets, modeling approaches, validation strategies, and evaluation metrics, a meta-analysis was not conducted. Instead, the evidence was synthesized using descriptive synthesis and narrative synthesis. Descriptive synthesis was used to summarize study characteristics, educational contexts, prediction targets, model families, predictor categories, feature selection methods, explainability techniques, optimization procedures, and evaluation metrics. Narrative synthesis was used to identify methodological patterns, limitations, and research gaps across the included studies.

The synthesis was organized according to the four research questions. For RQ1, the analysis summarized the machine learning and predictive modeling approaches used across the included studies. For RQ2, the synthesis classified the predictors used in the models and identified those reported as relevant by the original studies. For RQ3, the synthesis examined how feature selection, dimensionality reduction, hyperparameter optimization, explainability methods, and bio-inspired optimization were incorporated into predictive pipelines. For RQ4, the synthesis identified methodological gaps and future research opportunities related to validation, reproducibility, interpretability, metaheuristic-based feature selection, and institutional deployment.

The eligibility of studies for each synthesis was determined according to their relevance to the corresponding research question and the availability of extractable information in the published reports. Studies could contribute to more than one synthesis when they reported evidence relevant to multiple analytical dimensions, such as model comparison, predictor relevance, feature selection, explainability, hyperparameter optimization, or bio-inspired optimization. When information was missing, ambiguous, or insufficiently described, it was coded as ‘Not reported’ or ‘Unclear’ and was not inferred beyond what was explicitly available in the original publication.

Findings were reported using summary tables and narrative comparison. Tables were used to organize the characteristics of individual studies, prediction targets, model families, predictor categories, evaluation metrics, methodological quality criteria, and pipeline components. Narrative comparison was used to synthesize cross-study patterns and to interpret differences in relation to study context, prediction formulation, dataset characteristics, validation strategy, and reporting completeness. Claims about model performance, predictor relevance, or methodological benefits were limited to what was explicitly reported in the included studies.

No formal statistical assessment of reporting bias or certainty of evidence, such as funnel plot analysis or GRADE, was conducted because the review synthesized heterogeneous predictive modeling studies with diverse outcomes, datasets, validation strategies, and evaluation metrics, and no meta-analysis was performed. Instead, confidence in the evidence was discussed narratively through the methodological quality assessment, reporting completeness, validation practices, limitations of the included studies, and the consistency of methodological patterns observed across the corpus.

## 3. Results

### 3.1. Study Selection and Corpus Description

The database search identified 278 records, including 135 records from Web of Science Core Collection and 143 records from Scopus. After removing 99 duplicate records, 179 unique records were retained for title and abstract screening. Of these, 141 records were excluded because they did not meet the predefined eligibility criteria. The remaining 38 reports were sought for full-text assessment, and all were successfully retrieved. After full-text assessment, 13 reports were excluded, resulting in a final corpus of 25 studies included in the synthesis.

**Figure 1:**
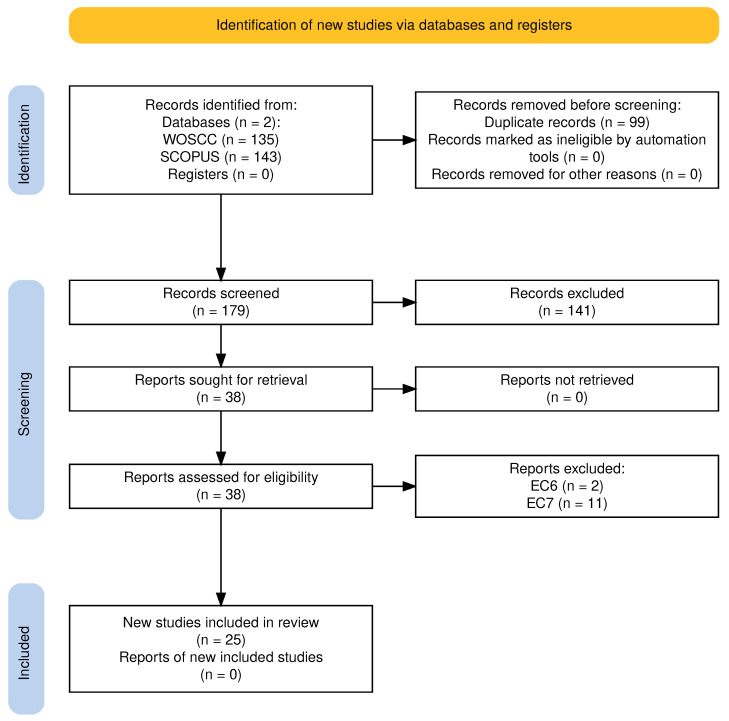
PRISMA 2020 flow diagram summarizing the study selection process.

The final corpus reflects the specific scope of this review, which focused on predictive or computational approaches explicitly addressing timely graduation, on-time graduation, time-to-degree, degree completion, student completion, completion within an expected period, or directly equivalent outcomes in higher education. Figure 1 summarizes the study selection process following the PRISMA 2020 flow structure. The identification stage retrieved 278 records from Web of Science Core Collection and Scopus. After removing 99 duplicates, 179 unique records were screened by title and abstract, leading to the exclusion of 141 records that did not meet the predefined eligibility criteria. The main reasons for exclusion at this stage were explanatory or associative statistical modeling without predictive evaluation, non-predictive or non-empirical study design, ineligible population, and outcomes limited to course, exam, GPA-only, or first-year progression measures. Full-text assessment was then conducted for 38 reports, all of which were retrieved. At this stage, 13 reports were excluded because they used exploratory unsupervised approaches without supervised predictive validation or statistical/econometric models focused on impact estimation, causal inference, or retrospective association analysis without predictive evaluation. Consequently, 25 studies were included in the final qualitative synthesis. The distribution of title and abstract exclusion reasons is presented in Table 1, while full-text exclusion reasons are summarized in Table 2. The complete screening records, eligibility decisions, and exclusion reasons are provided in Supplementary File S1.

The relatively high exclusion rate after title and abstract screening should be interpreted in light of the deliberately sensitive initial search strategy and the narrow conceptual focus of this review. The search was designed to retrieve a broad set of records potentially related to graduation, completion, educational prediction, and computational modeling. However, the eligibility criteria were intentionally restrictive at the screening stage to retain only studies that explicitly modeled graduation-related or completion-related outcomes in higher education using predictive or computational approaches. Many excluded records addressed adjacent student-success outcomes, explanatory statistical associations, course-level performance, or non-predictive educational analyses. Therefore, the exclusion rate does not necessarily indicate an overly restrictive search strategy, but rather reflects the distinction between the broad retrieval strategy and the focused analytical scope required for a review centered on graduation prediction.

### 3.2. Descriptive Overview of the Included Studies

This section provides a descriptive and quantitative overview of the 25 studies included in the final corpus. The analysis summarizes publication trends, geographical distribution, institutional scope, sample-size characteristics, target outcomes, and predictive formulations. Table 3 provides a study-level summary of the included corpus.

#### 3.2.1. Number of Studies by Publication Year

**Figure 2:**
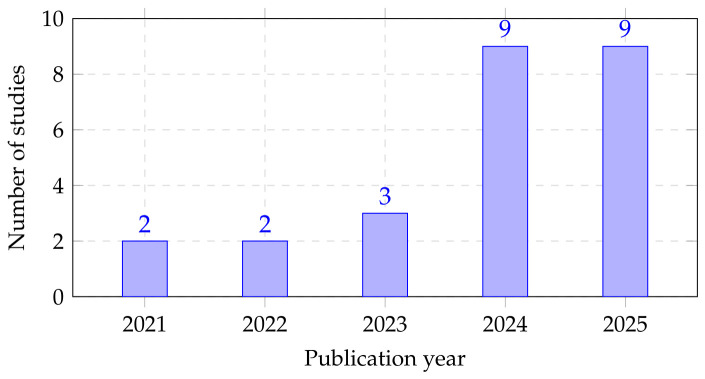
Distribution of included studies by publication year.

Figure 2 presents the annual distribution of the included studies. The corpus shows limited publication activity between 2021 and 2023, with two studies published in 2021 [23,48], two in 2022 [37,42], and three in 2023 [31,33,47]. In contrast, publication activity increased in the two most recent years covered by the review, with nine studies published in 2024 and nine in 2025. Overall, 18 of the 25 included studies were published during 2024–2025 [7,8,9,12,19,20,32,34,35,36,38,39,40,41,43,44,45,46].

This distribution suggests that predictive modeling for on-time graduation, degree completion, and related student completion outcomes has received greater attention in the most recent period analyzed. Nevertheless, this pattern should be interpreted with caution, as it reflects the composition of the final corpus rather than the complete evolution of the broader research field.

#### 3.2.2. Number of Studies by Country or Region

**Figure 3:**
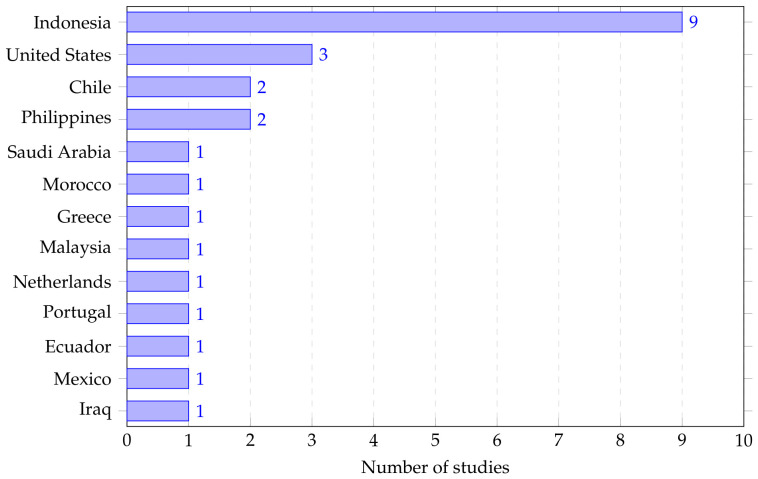
Distribution of included studies by country or region.

Figure 3 summarizes the distribution of the included studies by country or regional context. The studies were distributed across 13 countries or regions, although the corpus was not evenly represented geographically. Indonesia accounted for the largest number of studies, with nine contributions [8,20,39,41,43,44,45,46,47]. The United States contributed three studies [9,12,36], while Chile [34,37] and the Philippines [40,48] contributed two studies each. The remaining studies were distributed across Saudi Arabia [7], Morocco [31], Greece [23], Malaysia [32], the Netherlands [33], Portugal [35], Ecuador [19], Mexico [38], and Iraq [42], with one study in each case.

This distribution suggests that research on predictive modeling for timely graduation and related completion outcomes has been conducted in diverse higher education contexts. However, the concentration of studies in a limited number of countries, particularly Indonesia, also indicates that the available evidence may be influenced by context-specific institutional data structures, educational systems, and reporting practices. Therefore, cross-country comparability should be interpreted cautiously.

#### 3.2.3. Number of Studies by Journal or Source

Table 4 reports the distribution of the included studies by journal or source. The 25 studies were published across 25 distinct peer-reviewed journals and proceedings, with each outlet contributing one study to the final corpus. Therefore, no dominant publication venue was identified within the reviewed set.

This pattern suggests a highly dispersed publication landscape for research on timely graduation prediction and related completion outcomes. Rather than being concentrated in a small group of specialized journals, the topic appears across outlets associated with educational technology, applied computer science, engineering education, data science, policy analysis, learning analytics, and applied analytics. The fact that each source contributed only one study indicates that the field has not yet consolidated around a stable set of core publication venues. This dispersion may reflect the interdisciplinary nature of graduation-related prediction, which sits at the intersection of higher education research, educational data mining, institutional analytics, and machine learning. However, it may also limit cumulative knowledge building, because relevant studies are scattered across disciplinary communities with different methodological expectations, terminology, and evaluation standards.

The absence of recurrent publication venues also has implications for future reviews and bibliometric monitoring. Because the literature is scattered across heterogeneous sources, search strategies should combine higher education, educational analytics, and computational keywords rather than relying only on a small set of specialized journals. As the field matures, the emergence of more recurrent publication venues could facilitate stronger theoretical integration, methodological comparability, and cumulative debate around graduation prediction models.

#### 3.2.4. Number of Studies by Institutional Scope

Table 5 presents the distribution of the included studies by institutional scope. Most studies relied on data from a single institution, with 24 studies using records from one university or an equivalent higher education institution. Only one study used a broader multi-institutional or system-level administrative dataset.

This pattern suggests that predictive modeling for timely graduation and related completion outcomes has been primarily developed in institution-specific settings. While such datasets may support models tailored to local academic structures, student populations, and administrative processes, they may also constrain the extent to which findings can be generalized across institutions or higher education systems. Consequently, further studies using multi-institutional or system-level data would be valuable for assessing the external validity and transferability of predictive models in this domain.

#### 3.2.5. Sample Size Range and Distribution

Table 6 categorizes the sample size information reported across the included studies. The number of student records ranged from a minimum of 111 students [34] to a maximum of 385,800 students [36]. With the sample sizes of all 25 studies fully reported, the median sample size across the corpus is 2184 students [8].

These results show considerable variation in the empirical scale of the reviewed studies. As observed in the distribution, several studies relied on relatively small, institution-specific datasets comprising fewer than 500 students, whereas others utilized much larger administrative datasets exceeding 10,000 records. This heterogeneity is relevant for interpreting model performance, as sample size may critically influence training stability, validation strategies, class imbalance handling, and the extent to which predictive findings can be generalized or compared across different institutional contexts.

Taken together, the institutional-scope and sample-size findings provide the main empirical basis for assessing scalability in the reviewed corpus. Although some studies used large administrative datasets, the evidence remains dominated by single-institution designs. Therefore, the reviewed literature provides stronger evidence of local model feasibility than of scalability across cohorts, programs, institutions, or higher education systems.

#### 3.2.6. Number of Studies by Target Outcome

**Figure 4:**
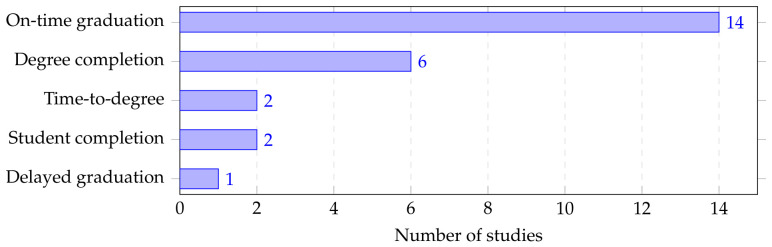
Distribution of included studies by target outcome.

Figure 4 presents the distribution of the included studies according to the target outcome addressed in the predictive task. The studies operationalized student success through conceptually related but distinct outcomes. The most frequent target outcome was on-time graduation, reported in 14 studies [7,8,9,31,32,34,38,39,41,43,44,45,46,47]. This was followed by degree completion, which appeared in six studies [12,33,36,40,42,48]. Less frequent outcome formulations included time-to-degree [23,35], student completion [19,20], and delayed graduation [37].

Overall, the distribution indicates that the reviewed literature has mainly framed graduation-related prediction as a task concerned with whether students complete their studies within an expected period. Fewer studies explicitly modeled time-to-degree or delayed graduation, suggesting that continuous or temporally explicit formulations remain less represented in the corpus. This distinction is methodologically relevant because different target definitions may require different modeling strategies, evaluation metrics, and interpretations of student progression.

#### 3.2.7. Number of Studies by Predictive Formulation

**Figure 5:**
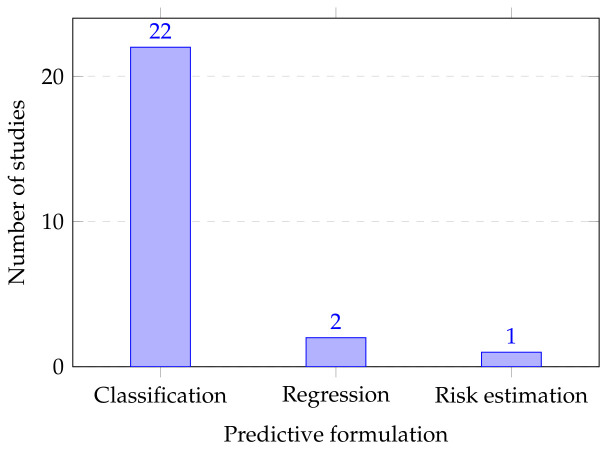
Distribution of included studies by predictive formulation. Although some studies used more than one modeling approach, each study was categorized according to its main predictive formulation.

Figure 5 presents the distribution of the included studies by predictive formulation. Most studies framed the prediction problem as a classification task, with 22 studies assigning students to discrete outcome categories, such as graduating on time versus not graduating on time, completing a degree versus not completing it, or related categorical formulations [7,8,9,12,19,20,31,32,33,34,36,38,39,40,41,42,43,44,45,46,47,48]. Two studies formulated the problem as a regression task to estimate time-to-degree more explicitly [23,35], whereas one study focused on risk estimation by calculating delay-related risk scores [37].

This distribution indicates that timely graduation and related completion outcomes have been mainly operationalized as classification problems. This framing is methodologically consistent with institutional decision-support settings in which students are commonly grouped according to expected completion status. However, the limited number of regression and risk-estimation studies suggests that temporally explicit or risk-oriented formulations remain less represented in the reviewed corpus.

### 3.3. RQ1: Machine Learning Approaches for Graduation Prediction

The reviewed studies show that graduation-related prediction in higher education has been approached mainly as a supervised learning problem. Most studies formulated the target as a classification task, including on-time graduation, delayed graduation, degree completion, or completion-related risk. A smaller group of studies framed the problem as regression, time-to-degree estimation, survival analysis, clustering-assisted prediction, or trajectory modeling. This methodological diversity reflects the different ways in which graduation-related outcomes have been operationalized across institutional contexts.

Table 7 summarizes the main model families identified in the reviewed corpus. Tree-based and ensemble methods were the most frequently reported family, followed by traditional machine learning algorithms, neural networks or deep learning models, and statistical predictive models. Survival analysis and clustering methods appeared less frequently and were generally used to incorporate temporal information or identify student subgroups before predictive modeling. Because several studies evaluated or compared multiple algorithms, the counts reported in Table 7 are not mutually exclusive.

Tree-based and ensemble methods were the most common approaches in the corpus. Random Forest, decision trees, XGBoost, AdaBoost, LightGBM, and bagging-based methods were used either as primary models or as comparators in multiple studies, as summarized in Table 7. Within this group, Random Forest, decision tree variants, and boosting methods were the most recurrent algorithmic choices. These methods were frequently selected because they can model nonlinear relationships and interactions among academic, demographic, institutional, and behavioral variables. Several comparative studies reported Random Forest or boosting-based algorithms among the best-performing alternatives, although the reported performance varied according to the prediction target, data structure, validation strategy, and evaluation metric.

Traditional machine learning models, including Support Vector Machines, Naive Bayes, and K-Nearest Neighbors, were also commonly used, mainly in comparative experimental designs or as baseline classifiers. Their reported performance ranged from moderate to high across studies, but no single traditional classifier consistently outperformed the alternatives across all contexts.

Neural network and deep learning approaches were less frequent than tree-based and traditional machine learning models, but they were used in several studies focused on on-time graduation or completion prediction. These models were generally applied to capture nonlinear patterns in student data. However, their use was usually accompanied by the need for careful validation and, in some cases, additional interpretability mechanisms, given their more opaque internal structure.

Statistical predictive models remained relevant in the reviewed literature. Logistic regression, multiple linear regression, and penalized multinomial regression were used either as predictive models or as interpretable baselines. These approaches were especially useful when studies aimed to combine prediction with direct interpretation of coefficients, odds ratios, or variable effects. Their inclusion also suggests that graduation prediction research has not moved exclusively toward complex machine learning models; several studies continue to use statistical models when interpretability is a priority.

A smaller number of studies used survival analysis or clustering-assisted approaches. For example, ref. [38] used Cox proportional hazards modeling to incorporate the temporal dimension of student academic trajectories. Clustering-assisted approaches, including K-Means and Gaussian finite mixture models, were used to identify latent student groups or trajectory profiles before prediction [23,37]. These approaches were less common than supervised classification models, but they are relevant because timely graduation and time-to-degree are inherently temporal and progression-based outcomes.

Finally, ref. [32] incorporated Quantized Embeddings Reduction (QED) as part of a predictive pipeline involving high-dimensional representations. This type of approach was not common in the corpus, but it illustrates the emergence of methods designed to reduce representation complexity before applying machine learning classifiers.

### 3.4. RQ2: Predictors Used in Timely Graduation and Time-to-Degree Models

The reviewed studies used a broad range of predictors to model timely graduation, time-to-degree, degree completion, and related completion outcomes. These predictors were grouped into eight categories: academic performance, sociodemographic variables, pre-college or admission variables, socioeconomic or financial variables, institutional variables, curricular or program-level variables, behavioral variables, and longitudinal academic trajectory variables. Table 8 summarizes the predictor categories identified in the corpus, including representative variables, number of studies, included studies, and the methods used to establish predictor relevance.

As shown in Table 8, academic performance was the most frequently used predictor category. Variables such as cumulative GPA, semester GPA, credits earned, failed courses, and academic progression indicators appeared in 23 studies. These variables were often reported as relevant through feature importance, permutation feature importance, correlation analysis, SHAP values, or decision rules. This pattern suggests that models for timely graduation and time-to-degree prediction rely heavily on academic trajectory information once students have accumulated sufficient institutional records.

Pre-college and admission variables were also commonly used, appearing in 15 studies. High school GPA, entrance examination scores, and high school type or accreditation were generally used as early indicators of student readiness. These variables are particularly relevant for early-warning settings, where university-level performance data may not yet be available. However, some studies indicated that admission-related variables alone may provide weaker predictive signals than academic performance accumulated during the degree program; for example, ref. [7] reported that admission data performed poorly compared with academic variables.

Sociodemographic and socioeconomic variables were frequently incorporated to contextualize student profiles and identify differential risk patterns. Age, gender, ethnicity or race, nationality, domicile, family income, parental education, scholarships, and financial aid were among the most common variables in these categories. Their relevance was usually assessed through statistical significance, correlation analysis, SHAP, LIME, or feature importance methods. These variables require careful interpretation because they may reflect structural inequalities rather than directly actionable academic factors. In this regard, ref. [36] highlighted the importance of examining algorithmic calibration and fairness when demographic variables are included or omitted from predictive models.

Institutional, curricular, and program-level variables provided additional context for explaining differences in completion patterns across programs, campuses, departments, and graduation modalities. Variables such as major, specialization, study plan hours, course difficulty, graduation modality, and advisor-related information were used to capture structural aspects of academic progression. For instance, studies focused on time-to-degree and late completion used variables such as years in degree, graduation modality, program-level median time-to-degree, and time since first enrollment to represent academic trajectory and institutional structure [34,35].

Behavioral and longitudinal variables were less common but relevant for specific predictive tasks. Class attendance, study time, extracurricular participation, application timing, family support, and temporal progression indicators were used to capture aspects of engagement and student trajectory that are not fully reflected in grades. Although these categories appeared in fewer studies, they may improve the contextual interpretation of student risk when combined with academic and institutional variables. Overall, the reviewed evidence indicates that predictor selection in graduation-related models is concentrated around academic performance, but more comprehensive models often combine academic, sociodemographic, socioeconomic, institutional, curricular, behavioral, and longitudinal information.

### 3.5. RQ3: Feature Selection, Explainability, and Optimization in Predictive Pipelines

The third research question examined how the included studies incorporated feature selection, dimensionality reduction, hyperparameter optimization, explainability, and bio-inspired optimization within graduation-related prediction pipelines. These components were analyzed as indicators of pipeline maturity because they address different methodological needs: feature selection and dimensionality reduction reduce the input space, hyperparameter optimization adjusts model configuration, explainability supports interpretation, and bio-inspired optimization provides search-based mechanisms for improving model design, tuning, or variable selection. For clarity, bio-inspired optimization was coded only when a study explicitly used a nature-inspired or metaheuristic algorithm as an active component of the predictive pipeline, such as for feature selection, hyperparameter optimization, model configuration, or related search tasks. General use of machine learning models was not coded as bio-inspired unless such an optimization mechanism was explicitly reported. The targeted coding distinguished between active implementation, background-only mention, absence, and unclear reporting of bio-inspired or metaheuristic methods. Active implementation was identified in two studies: [20], which used Particle Swarm Optimization for model optimization, and [19], which implemented Genetic Algorithms and Ant Colony Optimization for feature selection. One additional study mentioned a bio-inspired method only as background literature, without implementing it in the predictive pipeline [12]. The remaining 22 studies did not mention bio-inspired or metaheuristic methods in the article text. Therefore, the claim that bio-inspired optimization is underutilized is supported by the distribution of evidence across the reviewed corpus: two studies actively implemented these methods, one mentioned them only as background, and 22 did not report them.

Overall, 20 of the 25 included studies explicitly reported feature selection, while three reported dimensionality reduction procedures. These categories were not treated as mutually exclusive. Feature selection was the most frequently reported pipeline component, followed by explainability or interpretability methods, which appeared in 17 studies, and hyperparameter optimization, which appeared in 14 studies. In contrast, active implementation of bio-inspired optimization was identified in only two studies. This contrast indicates that, although graduation prediction pipelines increasingly incorporate model refinement and interpretation procedures, the use of metaheuristic and bio-inspired algorithms remains marginal in the reviewed corpus.

Table 9 disaggregates these methodological components and reports their specific adoption across the corpus.

Feature selection was widely reported across the included studies. The methods used varied substantially, ranging from simple filter-based procedures to embedded and wrapper-based approaches. Filter methods included correlation analysis, ReliefF, Gain Ratio, and related statistical criteria. Embedded methods were mainly based on tree-derived importance measures, such as mean decrease in impurity, or on permutation feature importance. Wrapper-based approaches were less common, with BorutaShap appearing as a more computationally demanding feature selection strategy in a high-dimensional setting. Overall, feature selection was used to reduce the input space, identify relevant predictors, and support model interpretation, although the level of methodological detail varied across studies.

Dimensionality reduction was less frequently reported than feature selection. The identified methods included clustering-based reduction, Principal Component Analysis, and Quantized Embeddings Reduction. For example, ref. [32] used Quantized Embeddings Reduction to address high-dimensional representations derived from language-model-based embeddings. This type of approach was uncommon in the corpus, but it illustrates how dimensionality reduction may become relevant when predictive pipelines incorporate richer or more complex educational data representations.

Hyperparameter optimization was reported in more than half of the included studies. The most common approaches were grid search, GridSearchCV, random search, manual tuning, and Optuna. These methods were used to adjust model configurations for classifiers such as Random Forest, Support Vector Machines, neural networks, and ensemble models. In most cases, hyperparameter optimization was implemented through conventional search procedures rather than through metaheuristic optimization. One exception was [20], which actively used Particle Swarm Optimization for hyperparameter tuning.

Explainability and interpretability methods were also frequently incorporated into the predictive pipelines. Some studies relied on intrinsically interpretable outputs, such as regression coefficients, decision rules, or statistical significance. Others applied post hoc methods, including SHAP, LIME, permutation feature importance, and tree-based feature importance. These methods were used to identify influential variables, support model interpretation, and make predictions more understandable for educational decision-making. For instance, SHAP and LIME were used in several studies to provide global and local explanations for more complex models [7,9,12].

Bio-inspired optimization and metaheuristics were rarely incorporated as active components of graduation-related prediction pipelines. The targeted coding distinguished between active implementation, background-only mention, absence, and unclear reporting of bio-inspired or metaheuristic methods. Active implementation was identified in two studies: [20], which used Particle Swarm Optimization for model optimization, and [19], which implemented Genetic Algorithms and Ant Colony Optimization for feature selection. One additional study mentioned a bio-inspired method only as background literature, without implementing it in the predictive pipeline [12]. The remaining 22 studies did not mention bio-inspired or metaheuristic methods in the article text. This distribution supports a precise interpretation of underutilization: within the reviewed corpus, bio-inspired optimization was not absent, but its use was limited to isolated pipeline tasks rather than being systematically evaluated as a broader modeling or decision-support strategy for graduation prediction.

Within the reviewed corpus of 25 studies, no study explicitly formulated graduation prediction as a multi-objective optimization problem. This finding should therefore be interpreted as an observation from the analyzed evidence base rather than as a definitive claim about the entire field of educational data mining or higher education analytics. The absence of this formulation is methodologically relevant because timely graduation prediction is not only a classification or regression problem, but also a decision-support problem in which institutional usefulness depends on trade-offs among predictive performance, transparency, equity, feasibility, computational cost, and operational deployment. From a bio-inspired perspective, these trade-offs are precisely the type of search and optimization setting in which population-based metaheuristics may provide value, particularly for feature subset selection, hyperparameter tuning, model simplification, threshold calibration, and fairness-aware optimization. Consequently, the limited adoption of bio-inspired and multi-objective optimization constitutes a key transition point from the current evidence base toward future scalable Bio-Inspired Educational Analytics, where metaheuristic search may support feature reduction, efficient model tuning, model simplification, and trade-off analysis under institutional and computational constraints.

### 3.6. Results of the Methodological Quality Assessment

**Figure 6:**
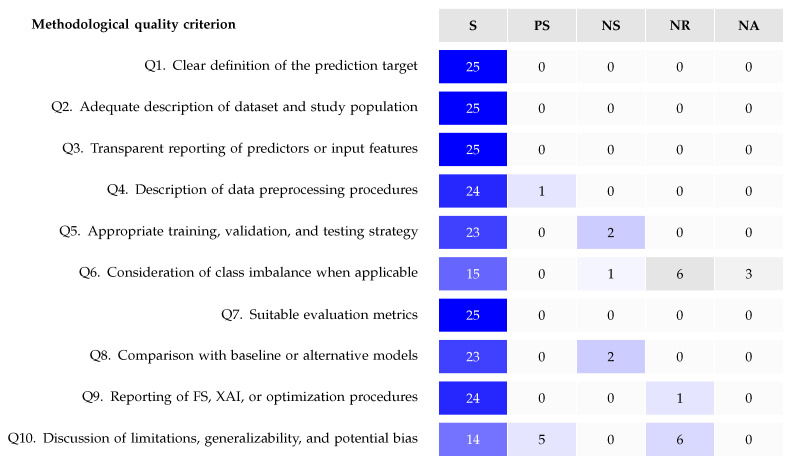
Heatmap of methodological quality codes across assessment criteria. Cell values indicate the number of studies (n=25). S = satisfied; PS = partially satisfied; NS = not satisfied; NR = not reported; NA = not applicable. Darker blue cells indicate higher counts.

A methodological quality assessment was conducted to evaluate the transparency, reliability, and reproducibility of the 25 included studies. The assessment was based on the ten criteria defined in the methodology: prediction target definition, dataset and population description, predictor reporting, preprocessing, validation strategy, class imbalance consideration, evaluation metrics, baseline or alternative model comparison, reporting of feature selection, explainability or optimization procedures, and discussion of limitations, generalizability, or potential bias. Each criterion was coded as satisfied (S), partially satisfied (PS), not satisfied (NS), not reported (NR), or not applicable (NA). Figure 6 provides a visual summary of the aggregate counts, and Table 10 presents the corresponding study-level assessment. This structure allows readers to interpret both the overall reporting patterns and the specific studies associated with each methodological strength or weakness.

Table 10 complements the aggregate summary and the visual distribution presented in Figure 6 by identifying the specific studies associated with each quality code. Overall, the reviewed studies reported the core components required for predictive modeling research. All 25 studies clearly defined their prediction targets, described their datasets or populations, reported their predictors or input features, and provided evaluation metrics aligned with their predictive formulation. Preprocessing was also generally well reported, with 24 studies satisfying this criterion and one study partially satisfying it.

The study-level assessment also makes visible several methodological weaknesses that are less evident from aggregate counts alone. Two studies did not satisfy the validation criterion because their evaluation procedures provided limited evidence of an appropriate independent or out-of-sample assessment [39,47]. Class imbalance was the least consistently addressed criterion: 15 studies explicitly addressed it through procedures such as resampling, weighting, imbalance-aware evaluation, or explicit class-ratio assessment; six studies did not report any treatment; one study did not satisfy the criterion because the imbalanced distribution was retained and associated with weak minority-class sensitivity [38]; and three studies were coded as not applicable because their prediction formulation did not correspond to a conventional imbalanced classification setting.

Comparison with baseline or alternative models was reported in most of the corpus. Twenty-three studies compared multiple algorithms, model configurations, or reference approaches, whereas two studies relied on a single model without an adequate comparison. Similarly, 24 studies reported at least one relevant pipeline component, including feature selection, dimensionality reduction, explainability, hyperparameter optimization, or related methodological procedures. Examples include automated hyperparameter tuning frameworks such as Optuna [8,41] and explainability techniques such as SHAP and LIME [7,12]. This indicates that most studies went beyond simple model fitting, although the depth and transparency of reporting varied across articles.

The weakest reporting dimension was the discussion of limitations, generalizability, and potential bias. This criterion was fully satisfied in 14 studies, partially satisfied in five, and not reported in six. This pattern is visible in Figure 6, where Q10 concentrates several partially satisfied and not reported codes. A stronger treatment of fairness and calibration was observed in [36], which explicitly examined subgroup calibration issues and algorithmic bias. This finding is relevant because most models were developed using institution-specific datasets, and several studies relied on historical records without external validation or deployment evaluation. Therefore, while the corpus shows relatively strong reporting of model construction and evaluation procedures, the evidence base remains more limited regarding external validity, bias assessment, and the conditions under which predictive models could be responsibly transferred or implemented in institutional decision-making contexts.

### 3.7. Descriptive Synthesis of Reported Evaluation Metrics

Because the included studies used heterogeneous prediction targets, task formulations, validation designs, and evaluation metrics, model performance was synthesized descriptively rather than through statistical meta-analysis or pooled performance estimates. The extraction considered the prediction task type, prediction target, reported metrics, main performance values when explicitly available, validation strategy, and comparability limitations for each study. Table 11 summarizes the main families of evaluation metrics reported across the corpus. It does not compare model performance across studies; rather, it summarizes the heterogeneity of evaluation practices in the reviewed corpus.

This descriptive synthesis reinforces the decision not to calculate pooled performance estimates. The studies differed substantially in their outcome definitions, including binary on-time graduation, delayed graduation, degree completion, multiclass academic trajectories, time-to-degree estimation, continuous risk estimation, and survival-oriented formulations. They also differed in validation strategy, ranging from cross-validation and holdout designs to studies with limited or unclear out-of-sample assessment. Finally, the reported metrics were not consistent across the corpus: some studies reported threshold-dependent metrics such as accuracy, precision, recall, and F1-score, whereas others reported AUC, C-statistic, regression errors, survival metrics, or imbalance-aware indicators. Pooling these estimates would therefore combine non-equivalent prediction targets, task formulations, validation procedures, metric types, and outcome scales, producing a statistically misleading summary of model performance.

### 3.8. RQ4: Methodological Gaps and Future Research Opportunities

Based on the synthesis of the included studies and the methodological quality assessment reported in Section 3.6, several methodological gaps and future research opportunities were identified. These gaps concern the development, interpretation, validation, optimization, and institutional use of predictive models for timely graduation and related completion outcomes. They were derived from patterns observed across the corpus, limitations explicitly reported in the reviewed studies, and recurrent issues identified in the quality assessment, particularly those related to bio-inspired optimization, explainability, fairness, validation, class imbalance, external validity, and institutional deployment. Table 12 summarizes the main gaps, supporting evidence, representative studies, methodological implications, and future research directions.

As shown in Table 12, the reviewed corpus indicates that bio-inspired optimization and metaheuristics have been used only in specific pipeline tasks. Ref. [20] used Particle Swarm Optimization for hyperparameter tuning, whereas ref. [19] implemented Genetic Algorithms and Ant Colony Optimization for feature selection. In the latter case, the final methodological choice favored a filter-based method due to efficiency and performance considerations. This pattern suggests that the contribution of metaheuristics to graduation prediction remains at an early stage and has not yet been systematically assessed across different datasets, model families, and optimization objectives.

The limited use of bio-inspired methods is especially relevant because graduation prediction is not only a supervised learning problem, but also a pipeline optimization and decision-support problem. Predictive models in this domain must often balance competing objectives, including accuracy, minority-class recall, interpretability, fairness, computational cost, model sparsity, and institutional actionability. However, no study in the reviewed corpus explicitly formulated graduation prediction as a multi-objective optimization problem. This absence opens a specific opportunity for Bio-Inspired Educational Analytics, where metaheuristic algorithms could be used to search for model configurations or feature subsets that provide acceptable trade-offs between predictive performance and responsible institutional use.

The evidence also indicates that explainability has been incorporated unevenly. Several studies reported global feature importance or model-based interpretability, but fewer provided local explanations for individual predictions. Studies using SHAP or LIME illustrate how global and case-specific explanations can complement aggregate model performance metrics [7,9,12]. This pattern points to a potential research direction focused on combining local explainability with feature selection and model simplification strategies, so that predictive outputs can better support academic advising when individual students are identified as being at risk of delayed graduation or non-completion.

Another gap concerns algorithmic fairness and subgroup performance. Only a limited number of studies explicitly examined whether predictive models behave differently across sociodemographic groups. For example, ref. [36] examined calibration-related issues across racial groups, showing the relevance of fairness analysis in higher education prediction. This pattern indicates that model evaluation in this field could be strengthened by incorporating subgroup validation, calibration analysis, and fairness-aware metrics alongside aggregate performance indicators. From a bio-inspired optimization perspective, this also suggests the need for future models that optimize not only global predictive performance, but also fairness-related objectives and subgroup robustness.

The reviewed literature also shows a gap between offline predictive modeling and institutional implementation. Most studies evaluated models using historical datasets, while evidence of live deployment, integration into academic information systems, or evaluation of actual interventions remains limited. This limits the extent to which predictive performance can be connected to changes in advising processes, student support, or graduation outcomes. This pattern points to a need for implementation-oriented studies that evaluate predictive systems under real institutional conditions and assess their impact over time.

Finally, external validation and longitudinal modeling remain important areas for further development. Most studies relied on single-institution datasets, which may limit the generalizability of findings across different higher education systems. In addition, many models used static snapshots of student data rather than dynamic information on academic progression. Studies incorporating time-to-degree variables, time-dependent risk indicators, or survival-oriented approaches indicate the relevance of modeling graduation as a temporal process [34,35,38]. This pattern points to a potential research direction focused on multi-institutional and longitudinal designs that allow predictive models to be tested across contexts and updated as new student data become available.

## 4. Discussion

### 4.1. Principal Findings

This systematic literature review examined how machine learning and predictive modeling approaches have been used to predict timely graduation, time-to-degree, and degree completion in higher education. The final corpus was selective: from 278 records identified in WoSCC and Scopus, 25 studies met the eligibility criteria and were included in the synthesis. This selection process is relevant because many excluded records addressed student success, retention, dropout, or academic performance, but did not explicitly model graduation-related outcomes with a predictive purpose. Therefore, the resulting corpus reflects a narrower and more methodologically specific body of research focused on predictive modeling for completion-related outcomes rather than the broader literature on student persistence or academic achievement.

**Figure 7:**
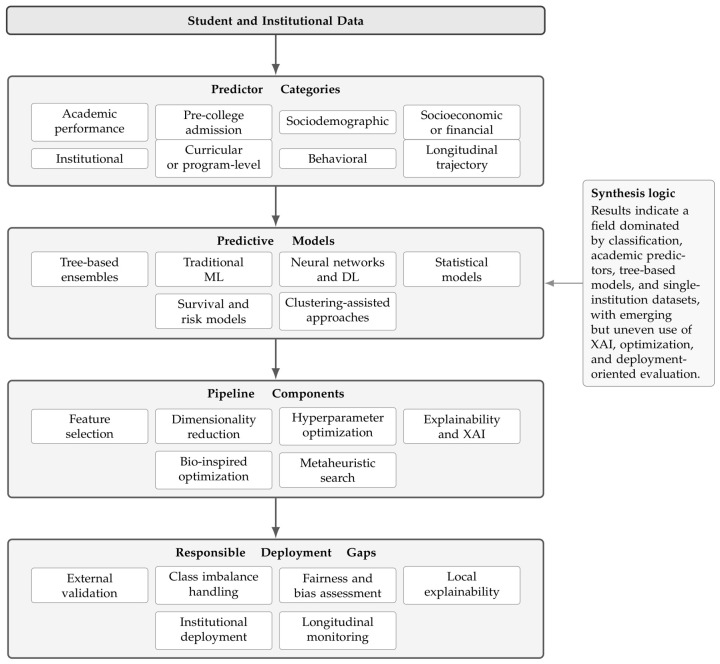
Conceptual synthesis of predictive modeling pipelines and methodological gaps in graduation-related prediction. The framework was developed by the authors based on the findings of the systematic review and should be interpreted as an integrative synthesis, not as a workflow empirically validated by the original studies.

As a synthesis of the evidence reported in Section 3, Figure 7 summarizes the main structure of graduation-related prediction research identified in this review. The figure represents the field as a pipeline that begins with student and institutional data, proceeds through predictor categories and predictive models, incorporates methodological pipeline components, and ends with unresolved responsible deployment gaps. This conceptual synthesis is not intended to prescribe a single modeling workflow. Rather, it summarizes the dominant patterns and methodological tensions observed across the reviewed corpus.

The findings indicate that this research area has expanded mainly in recent years, with most included studies published in 2024 and 2025. However, the corpus remains geographically and institutionally uneven. Indonesia accounted for nine of the 25 included studies, representing 36% of the reviewed corpus, followed by the United States with three studies. In addition, 24 of the 25 studies relied on single-institution datasets, whereas only one study used a multi-institutional or system-level dataset. This pattern indicates that the current evidence base is still dominated by local institutional contexts, a limitation that is discussed further in relation to scalability and external validity in Section 4.6.

Across the corpus, graduation-related prediction was predominantly formulated as a supervised classification problem. Most studies modeled whether students graduated on time, completed a degree, or belonged to a completion-related risk category. Fewer studies treated time-to-degree or delayed graduation as explicitly temporal, continuous, or risk-oriented outcomes. This distinction matters because classification models can support institutional early-warning systems, but they may simplify a process that unfolds over time and is affected by changing academic, socioeconomic, and institutional conditions.

This classification-oriented tendency provides the starting point for the following discussion, which first examines whether graduation-related prediction has been adequately modeled as a temporal progression problem.

### 4.2. Modeling Graduation as a Predictive and Temporal Problem

The predominance of classification reflects the operational needs of higher education institutions, where decision-makers often require categorical risk indicators to identify students who may need support. In this sense, binary or multiclass models of on-time graduation, degree completion, and delayed completion are compatible with advising workflows and student support systems. Nevertheless, this formulation may obscure the temporal structure of academic progression. Graduation is not a single static event but the endpoint of a sequence of academic decisions, course-taking patterns, institutional constraints, and student trajectories.

The limited number of studies using regression, risk estimation, survival analysis, or clustering-assisted approaches suggests that temporal modeling remains underdeveloped. Studies such as [34,35,38] illustrate the value of modeling time-to-degree, academic trajectory, or late completion more explicitly. These approaches can provide information that is not captured by simple on-time versus not-on-time classification. For example, time-to-degree estimation can distinguish students who are slightly delayed from those following more complex or prolonged trajectories.

This finding has methodological implications. If graduation-related prediction is framed only as classification, model evaluation may prioritize aggregate accuracy or F1-score without capturing how risk evolves across semesters. In contrast, time-to-event models, longitudinal predictors, and dynamically updated risk scores may better represent the progression-based nature of degree completion. Future studies should therefore consider whether their target definition matches the institutional decision being supported. A model designed for early academic advising may require different predictors, validation strategies, and evaluation metrics than a model designed to estimate final time-to-degree near the end of a program.

### 4.3. Predictor Relevance, Interpretability, and Institutional Actionability

The review found that academic performance was the most frequently used predictor category. Variables such as cumulative GPA, semester GPA, credits earned, failed courses, and study duration appeared in most studies and were frequently reported as relevant through feature importance, correlation analysis, SHAP values, permutation importance, or decision rules. This confirms that academic trajectory information remains central to graduation-related prediction once students have accumulated sufficient institutional records.

However, the centrality of academic performance also raises a practical limitation. Academic variables are usually strong predictors, but they may become available only after students have already experienced academic difficulty. For early-warning purposes, pre-college or admission variables, sociodemographic information, financial indicators, and institutional variables may be more useful at the beginning of the student lifecycle. The challenge is that these variables may provide weaker predictive signals than accumulated academic performance, and some may also reflect structural inequalities rather than directly modifiable student behaviors or institutional processes.

This creates a tension between predictive strength and actionability. Highly predictive variables are not always the most useful for intervention design. For example, GPA and credits earned can identify students at risk, but they do not necessarily explain which institutional action should be taken. In contrast, variables related to course bottlenecks, program structure, graduation modality, advising, financial aid, or time since first enrollment may offer more direct opportunities for institutional response. Studies incorporating institutional and curricular predictors therefore provide a useful direction for moving from risk detection toward actionable decision support.

The inclusion of sociodemographic and socioeconomic variables also requires careful interpretation. These variables can improve model performance and reveal differential risk patterns, but they may also encode historical inequities. The study by [36], which examined calibration and subgroup bias, is especially relevant in this regard. Predictive models in higher education should not only identify risk accurately but also avoid reinforcing inequitable assumptions about student potential. Consequently, predictor relevance should be interpreted jointly with fairness, explainability, and institutional responsibility.

### 4.4. Pipeline Maturity: From Model Fitting to Decision-Support Design

The results indicate that graduation-related prediction research is gradually moving beyond basic model fitting toward more structured predictive pipelines. Feature selection, explainability, and hyperparameter optimization were frequently reported, which suggests growing attention to model refinement, interpretability, and reproducibility. However, these components were not always integrated as part of a coherent decision-support design. In several studies, feature selection was mainly used to improve performance or reduce dimensionality, while explainability was often treated as a post hoc reporting tool rather than as a mechanism for guiding institutional action.

This distinction is important because predictive models for timely graduation are not only technical artifacts. They are potential decision-support instruments for academic advising, resource allocation, and student support. From this perspective, pipeline maturity should be evaluated not only by whether a study reports feature selection, model tuning, or explainability, but also by whether these components contribute to transparent, stable, and actionable decisions. A parsimonious model with well-justified predictors may be more useful for institutions than a more complex model with marginally higher performance but limited interpretability or weak deployment feasibility.

The limited implementation of bio-inspired optimization further shows that search-based pipeline design remains underdeveloped in this literature. As reported in the Section 3, only isolated studies used metaheuristics for model optimization or feature selection. Therefore, the main issue is not simply the absence of bio-inspired algorithms, but the lack of systematic evaluation of how such algorithms could support educational prediction pipelines under competing methodological and institutional requirements. This observation motivates the following discussion of Bio-Inspired Educational Analytics as a future research agenda rather than as an already consolidated practice.

### 4.5. Bio-Inspired Opportunities for Educational Analytics

The limited active implementation of metaheuristics in the reviewed corpus suggests that timely graduation prediction remains an underexplored application domain for biomimetics and bio-inspired computation. This gap is relevant because graduation-related prediction is not only a supervised learning task; it is also a pipeline optimization and decision-support problem. Educational datasets often include heterogeneous, redundant, and context-dependent variables, while institutions require models that are accurate, understandable, efficient, and feasible to implement. In this setting, bio-inspired algorithms should be understood as optimization mechanisms that can support the design and tuning of predictive pipelines, rather than as replacements for machine learning models.

A first opportunity concerns feature subset selection. Evolutionary and swarm-based algorithms can search for compact groups of predictors that preserve predictive performance while reducing redundancy and model complexity. This is particularly relevant in higher education, where smaller and more stable feature sets may improve transparency and facilitate communication with advisors, program directors, and institutional decision-makers. Unlike purely performance-oriented feature selection, bio-inspired approaches could be designed to favor predictors that are not only informative but also interpretable and feasible for institutional intervention.

A second opportunity concerns hyperparameter optimization and model simplification. Conventional tuning procedures such as grid search, random search, and automated search frameworks were more common in the reviewed corpus than metaheuristic alternatives. However, bio-inspired search may be useful when predictive pipelines involve large configuration spaces, high-dimensional data, or several competing design requirements. In these cases, algorithms such as Particle Swarm Optimization, Genetic Algorithms, Ant Colony Optimization, or Differential Evolution can explore candidate configurations without relying on exhaustive enumeration. Their potential value is especially relevant when model tuning must consider not only accuracy, but also computational efficiency, model parsimony, and operational feasibility.

A third opportunity concerns multi-objective educational analytics. Graduation prediction should not be optimized only for aggregate accuracy, because institutional decisions may depend on several competing criteria. Bio-inspired multi-objective algorithms could support the search for trade-offs among predictive performance, minority-class sensitivity, model simplicity, subgroup robustness, and deployment feasibility. This perspective is important because a slightly less accurate but more transparent and stable model may be more appropriate for responsible institutional use than a black-box model optimized only for maximum performance.

Overall, Bio-Inspired Educational Analytics should be understood as a methodological agenda for designing predictive systems that are technically effective and institutionally usable. Future studies should therefore move from isolated applications of PSO, Genetic Algorithms, or Ant Colony Optimization toward systematic comparisons between conventional optimization procedures and lightweight, hybrid, or multi-objective metaheuristic approaches. Such work would clarify whether nature-inspired search can contribute to scalable, interpretable, and responsible graduation prediction pipelines.

### 4.6. Methodological Quality, Scalability, and Responsible Deployment

The methodological quality assessment showed strong reporting in core predictive modeling elements, including target definition, predictor reporting, evaluation metrics, dataset description, preprocessing, and validation strategy. This suggests that the included studies generally provide enough methodological information to understand the basic structure of their predictive tasks. However, the assessment also identified weaker areas that are central for responsible use in higher education.

Class imbalance was one of the main issues. Although 15 studies explicitly addressed imbalance, six did not report its treatment despite its potential relevance, and one study retained a severely imbalanced distribution that was associated with weak minority-class recall [38]. This is important because graduation-related prediction often involves asymmetric costs. Failing to identify delayed or at-risk students may have more serious institutional consequences than misclassifying students who are likely to complete on time. Therefore, aggregate metrics such as accuracy should be complemented with class-sensitive metrics, confusion matrices, recall, precision, F1-score, AUC, calibration, and subgroup-specific performance.

The assessment also identified limitations in validation and baseline comparison. Two studies were coded as not satisfying the validation criterion because they did not provide adequate out-of-sample evaluation [39,47]. Although most studies compared alternative models, isolated cases relied on a single algorithm without an adequate benchmark. These issues matter because high reported performance can be misleading when validation is weak, when data leakage is not controlled, or when comparisons are not sufficiently informative. Predictive modeling studies in this field should therefore prioritize transparent validation designs, preferably including cross-validation, temporal validation, or external validation when data availability permits.

#### Scalability of Graduation Prediction Models

Scalability is an important consideration for interpreting the practical relevance of graduation-related prediction models. Because this study synthesizes published evidence rather than training a new empirical prediction model, scalability was examined through the characteristics of the reviewed studies rather than through an author-conducted split of a single student-level dataset. Specifically, it was assessed at the level of the reviewed evidence base by examining dataset size, institutional scope, validation design, and deployment-related reporting across the included studies. This approach is appropriate for a review because the aim is not to demonstrate the scalability of one model, but to evaluate whether the existing literature provides evidence that graduation prediction pipelines can scale across cohorts, programs, institutions, or higher education systems.

The reviewed evidence suggests that scalability remains insufficiently demonstrated. Although some studies used large administrative datasets, several relied on relatively small and institution-specific samples. More importantly, 24 of the 25 included studies were based on single-institution datasets, whereas only one study used a multi-institutional or system-level dataset. This pattern limits the extent to which reported model performance, predictor importance, and methodological conclusions can be assumed to transfer across institutional contexts. Graduation requirements, grading systems, academic calendars, curricular structures, student populations, and administrative data schemas differ substantially across higher education systems. Therefore, a model that performs well in one institutional setting may not necessarily remain robust when applied to another program, institution, or country.

Scalability should therefore be understood in three complementary ways. Computational scalability refers to the ability of predictive pipelines to handle larger datasets, higher-dimensional feature spaces, repeated model updates, and operational constraints such as training time and computational cost. Institutional scalability refers to the ability of models to accommodate heterogeneous academic rules, data infrastructures, advising practices, and definitions of on-time completion. Methodological scalability refers to the use of validation designs capable of testing model robustness beyond the original development context, including temporal validation, cross-cohort validation, external validation, and multi-institutional evaluation. From this perspective, the reviewed corpus provides stronger evidence of local model feasibility than of broad scalability. Future studies should therefore report scalability-related evidence more explicitly, including dataset size, feature dimensionality, computational cost, update frequency, cross-cohort performance, external validation, and feasibility of deployment in real academic information systems.

A related concern is the limited evidence of institutional deployment. Most studies were evaluated offline using historical datasets, with little evidence that models were integrated into academic information systems, advising workflows, or intervention protocols. This limits the practical interpretation of predictive performance. A model may perform well retrospectively but still fail to improve student outcomes if it is not scalable, accepted by institutional users, regularly updated, or connected to feasible support mechanisms. Future research should therefore evaluate not only predictive accuracy but also implementation conditions, decision workflows, user trust, intervention effects, longitudinal monitoring, and unintended consequences.

From the perspective of the Special Issue, these limitations also indicate where bio-inspired optimization may contribute to scalable solutions. Metaheuristic feature selection, efficient hyperparameter search, and multi-objective optimization can help reduce feature dimensionality, control computational cost, and balance predictive performance with interpretability and institutional feasibility. Thus, from a bio-inspired systems perspective, scalability in graduation prediction should not be understood only as the ability to process larger datasets, but also as the ability to design predictive pipelines that remain robust, interpretable, computationally feasible, and transferable across educational contexts.

### 4.7. Implications for Future Research

The synthesis suggests several directions for future work. First, future studies should move toward longitudinal and temporally aware modeling. Since graduation and time-to-degree are progression-based outcomes, models that update predictions as new academic data become available may provide more useful support than static models trained on a single snapshot. Time-to-event approaches, dynamic risk scores, and semester-by-semester prediction frameworks could strengthen the alignment between model design and student progression.

Second, future research should improve external validity and scalability. As discussed above, the dominance of single-institution datasets limits the generalizability of findings across higher education systems. Multi-institutional studies, cross-country comparisons, cross-cohort validation, and external validation designs are needed to determine which predictors and models transfer across institutional contexts and which remain context-specific.

Third, future work should integrate fairness, explainability, and deployment evaluation as core components of predictive modeling rather than as optional additions. The findings indicate that demographic and socioeconomic variables are frequently used, but fairness and subgroup calibration are rarely assessed in detail. In addition, local explainability remains limited despite its relevance for individualized advising. Responsible institutional deployment requires models that are not only accurate, but also interpretable, fair, maintainable, and connected to real intervention capacity.

Finally, future research should consolidate Bio-Inspired Educational Analytics through empirical studies that compare conventional optimization procedures with lightweight, hybrid, and multi-objective metaheuristic alternatives. These studies should evaluate whether nature-inspired search improves not only predictive performance, but also feature reduction, model simplicity, computational efficiency, transferability, and deployment feasibility.

## 5. Conclusions

This systematic literature review synthesized recent evidence on machine learning and predictive modeling approaches for timely graduation, time-to-degree, and degree completion prediction in higher education. From 278 records identified in WoSCC and Scopus, 25 studies met the eligibility criteria and were included in the final synthesis. The review showed that the literature is still relatively concentrated, with most studies published in the last two years of the search period and with a strong reliance on single-institution datasets. This indicates a growing but still fragmented research area, where predictive models are often developed for local institutional contexts rather than validated across broader higher education systems. From a scalability perspective, the evidence base therefore provides stronger support for local model feasibility than for transferability across cohorts, programs, institutions, or national higher education systems.

The findings indicate that graduation-related prediction has been predominantly formulated as a supervised classification task. Tree-based and ensemble methods were the most frequently reported modeling family, followed by traditional machine learning algorithms, neural networks or deep learning models, and statistical predictive models. Although classification models are well aligned with institutional early-warning systems, the limited use of regression, survival analysis, risk estimation, and temporally dynamic approaches suggests that the temporal nature of graduation and time-to-degree remains insufficiently modeled.

Regarding predictors, academic performance variables were the most frequently used and most consistently reported as relevant. GPA, credits earned, failed courses, and academic progression indicators appeared as central predictors across the corpus. However, more comprehensive models also incorporated sociodemographic, socioeconomic, institutional, curricular, behavioral, admission-related, and longitudinal variables. This combination suggests that graduation prediction should not be reduced to academic performance alone. Instead, future predictive systems should consider how academic trajectories interact with institutional structures, student background, program requirements, and support conditions.

The review also showed that many studies incorporated methodological components beyond basic model fitting. Feature selection, explainability, and hyperparameter optimization were frequently reported, while dimensionality reduction appeared less often. In contrast, bio-inspired optimization and metaheuristics were rarely implemented as active components of predictive pipelines. Only two studies implemented these methods directly: Particle Swarm Optimization for model optimization and Genetic Algorithms or Ant Colony Optimization for feature selection.. This limited adoption is a central finding for the biomimetics-oriented contribution of this review. It suggests that timely graduation prediction remains an underexplored application domain for bio-inspired computation, particularly for feature subset selection, model tuning, model parsimony, and multi-objective trade-off analysis.

The methodological quality assessment showed strong reporting in core predictive modeling elements, including target definition, predictor reporting, evaluation metrics, and validation in most studies. Nevertheless, relevant weaknesses remain. Class imbalance was not consistently addressed, some studies lacked adequate out-of-sample validation or baseline comparison, and the discussion of limitations, generalizability, and potential bias was uneven. These issues are critical because predictive models for graduation-related outcomes may inform institutional decisions affecting student support, advising, and resource allocation. Therefore, future work should move beyond retrospective accuracy and incorporate external validation, fairness assessment, local explainability, deployment evaluation, and longitudinal monitoring.

Overall, this review contributes a structured synthesis of the current evidence on machine learning-based graduation prediction in higher education and identifies a specific opportunity for developing scalable Bio-Inspired Educational Analytics. The findings suggest that the field has progressed in model diversity, predictor use, and pipeline sophistication, but still requires stronger methodological standards for external validation, fairness, interpretability, scalability, and institutional implementation. Future studies should move beyond isolated uses of Particle Swarm Optimization, Genetic Algorithms, or Ant Colony Optimization and systematically evaluate lightweight, hybrid, and multi-objective metaheuristic approaches. Such approaches should not be assessed only in terms of predictive accuracy, but also in relation to feature reduction, computational cost, model simplification, interpretability, fairness, transferability, and institutional actionability. In this sense, timely graduation prediction represents a promising domain for biomimetics and bio-inspired optimization, especially when predictive models are designed as scalable, responsible, and operationally useful systems for educational decision-making.

### Limitations of the Review

This review has several limitations. First, the search was restricted to WoSCC and Scopus, articles written in English, and publications from 2021 to 2025. This decision improved consistency and ensured that the corpus consisted of recent peer-reviewed studies with sufficient methodological detail, but it may have excluded relevant studies indexed in other databases, published in other languages, or disseminated as conference papers, book chapters, dissertations, institutional reports, or preprints.

Second, the review focused specifically on predictive or computational studies addressing timely graduation, on-time graduation, time-to-degree, degree completion, student completion, or equivalent completion outcomes. As a result, studies centered exclusively on dropout, retention, persistence, academic achievement, GPA, engagement, or first-year progression were excluded when they did not explicitly model graduation or completion. This criterion strengthened conceptual alignment with the review objective, but it also narrowed the corpus and excluded adjacent literature that may provide useful insights into student progression.

Third, the synthesis depended on the information reported in the included articles. When studies did not provide sufficient detail on preprocessing, validation, class imbalance, feature selection, explainability, hyperparameter optimization, or limitations, these elements had to be coded as not reported or partially satisfied. Therefore, some methodological gaps identified in this review may reflect limitations in reporting rather than limitations in the actual procedures implemented by the original authors.

Fourth, a meta-analysis was not conducted because the included studies differed substantially in prediction targets, datasets, institutional contexts, sample sizes, predictors, modeling approaches, validation strategies, and evaluation metrics. Consequently, the review does not provide pooled performance estimates or statistical comparisons across algorithms. Instead, the synthesis emphasized descriptive and narrative comparison, which is appropriate for heterogeneous evidence but does not allow causal or quantitative claims about the superiority of specific model families.

Fifth, scalability was assessed through the characteristics reported in the reviewed studies rather than through an author-conducted empirical validation on new student-level datasets. This approach is consistent with the systematic review design, but it means that the review cannot directly test whether a specific predictive model scales across institutions, cohorts, or national higher education systems. Instead, scalability-related conclusions are based on reported evidence such as institutional scope, sample size, validation design, external validation, computational-cost reporting, and deployment-related information. Future empirical studies should therefore evaluate scalable graduation prediction pipelines using multi-institutional datasets, cross-cohort validation, external testing, and explicit computational-cost analysis.

Sixth, the biomimetics-oriented interpretation of the findings should be understood as a research agenda derived from the systematic synthesis, rather than as an experimental evaluation of bio-inspired algorithms or scalable bio-inspired systems. This review identified the limited adoption of metaheuristics and bio-inspired optimization in the included corpus and discussed future opportunities for Bio-Inspired Educational Analytics. However, it did not implement, benchmark, or compare Particle Swarm Optimization, Genetic Algorithms, Ant Colony Optimization, Differential Evolution, or other metaheuristic approaches on educational datasets. Therefore, claims regarding their potential value for feature selection, hyperparameter optimization, model simplification, multi-objective trade-off analysis, or scalable deployment should be interpreted as directions for future empirical research rather than as evidence of superior predictive performance in graduation-related prediction.

Finally, although the review included a methodological quality assessment, the assessment was designed to contextualize the evidence rather than to exclude studies or rank them. The conclusions should therefore be interpreted as a synthesis of reported methodological patterns across the corpus, not as a definitive evaluation of the technical validity, institutional usefulness, or practical deployability of each individual model.

## Figures and Tables

**Table 1:** Reasons for exclusion during title and abstract screening.

Code	Exclusion Reason	Count
**EC1**	Ineligible educational context	1
**EC2**	Ineligible population	18
**EC3**	Ineligible main outcome	7
**EC4**	Adjacent outcome without explicit link to graduation/completion	7
**EC5**	Absence of predictive modeling	5
**EC6**	Non-predictive or non-empirical study	29
**EC7**	Statistical model with an explanatory, causal, or associative purpose, without predictive evaluation	63
**EC8**	Outcome limited to a specific course, exam, GPA-only measure, or first-year progression	10
**EC9**	False positive outside the educational domain	1
**Total**		**141**

**Table 2:** Reasons for exclusion after full-text assessment.

Code	Exclusion Reason	Count
**EC6**	Exploratory unsupervised approaches, such as clustering or latent class analysis, without a supervised predictive modeling objective or predictive validation	2
**EC7**	Statistical or econometric models focused on impact estimation, causal inference, explanatory associations, or retrospective association analysis, without predictive evaluation	11
**Total**		**13**

**Table 3:** Summary characteristics of the 25 included studies.

Study	Country	Data Source	Sample Size	Target Outcome	Task Type	Main Model
[8]	Indonesia	Single institution	2184	On-time graduation	Classification	DNN
[7]	Saudi Arabia	Single institution	5883	On-time graduation	Classification	Random Forest, SVM
[31]	Morocco	Single institution	5236	On-time graduation	Classification	Random Forest
[23]	Greece	Single institution	1100	Time-to-degree	Regression, clustering-assisted prediction	Random Forest
[12]	United States	Single institution	5637	Degree completion	Classification	Random Forest
[32]	Malaysia	Single institution	4007	On-time graduation	Classification	QED + Classifiers
[33]	Netherlands	Single institution	27,643	Degree completion	Classification	Multinomial Reg., Random Forest
[34]	Chile	Single institution	111	On-time graduation	Classification	Random Forest
[35]	Portugal	Single institution	2047	Time-to-degree	Regression	Random Forest
[36]	United States	Multi-institutional	385,800	Degree completion	Classification	Random Forest
[19]	Ecuador	Single institution	6690	Student completion	Classification	XGBoost
[37]	Chile	Single institution	513	Delayed graduation	Risk estimation	SVM
[9]	United States	Single institution	7871	On-time graduation	Classification	Logistic Regression
[38]	Mexico	Single institution	421	On-time graduation	Classification	Multinomial Regression
[39]	Indonesia	Single institution	229	On-time graduation	Classification	Naive Bayes
[40]	Philippines	Single institution	3417	Degree completion	Classification	Decision Tree
[20]	Indonesia	Single institution	5660	Student completion	Classification	Neural Network
[41]	Indonesia	Single institution	810	On-time graduation	Classification	SMLOS (Stacking)
[42]	Iraq	Single institution	13,262	Degree completion	Classification	Bagging, Random Forest
[43]	Indonesia	Single institution	460	On-time graduation	Classification	K-NN
[44]	Indonesia	Single institution	356	On-time graduation	Classification	CoR-ANN
[45]	Indonesia	Single institution	160	On-time graduation	Classification	Ensemble Stacking
[46]	Indonesia	Single institution	4093	On-time graduation	Classification	Random Forest
[47]	Indonesia	Single institution	140	On-time graduation	Classification	Bagging CART
[48]	Philippines	Single institution	638	Degree completion	Classification	Naive Bayes

**Table 4:** Distribution of included studies by journal or source.

Journal or Source	Included Study
Journal of ICT Research & Applications	[8]
TEM Journal	[7]
International Journal of Advanced Computer Science and Applications	[31]
Education and Information Technologies	[23]
Journal of Marketing Analytics	[12]
International Journal of Computational Intelligence Systems	[32]
Autism	[33]
Computer Applications in Engineering Education	[34]
International Journal of Educational Technology in Higher Education	[35]
Journal of Policy Analysis and Management	[36]
Data	[19]
IEEE Revista Iberoamericana de Tecnologias del Aprendizaje	[37]
International Journal of Engineering Education	[9]
Array	[38]
International Journal of Computing and Digital Systems	[39]
Review of Computer Engineering Research	[40]
ICIC Express Letters, Part B: Applications	[20]
Journal of Applied Data Sciences	[41]
International Journal of Intelligent Engineering and Systems	[42]
Indonesian Journal of Electrical Engineering and Computer Science	[43]
ICIC Express Letters	[44]
Data and Metadata	[45]
Journal of Online Informatics	[46]
BAREKENG: Journal of Mathematics and Its Applications	[47]
International Journal of Modern Education and Computer Science	[48]

**Table 5:** Distribution of included studies by institutional scope.

Institutional Scope	Count	Included Studies
Single institution	24	[7,8,9,12,19,20,23,31,32,33,34,35,37,38,39,40,41,42,43,44,45,46,47,48]
Multi-institutional or system-level	1	[36]
Total	25	

**Table 6:** Distribution of sample sizes across the included studies.

Sample Size Range	Count	Included Studies
Less than 500 students	7	[34,38,39,43,44,45,47]
500 to 1000 students	3	[37,41,48]
1001 to 5000 students	6	[8,23,32,35,40,46]
5001 to 10,000 students	6	[7,9,12,19,20,31]
More than 10,000 students	3	[33,36,42]
Total	25	

**Table 7:** Model families used for graduation-related prediction.

Model Family	Algorithms or Models Reported	Studies	Included Studies	General Pattern
Statistical predictive models	Multiple linear regression, logistic regression, penalized multinomial regression	9	[7,8,9,31,32,33,36,37,38]	Used mainly as interpretable predictive models or baseline approaches.
Traditional machine learning	SVM, Naive Bayes, KNN	16	[7,8,12,19,20,31,32,34,35,37,39,41,43,45,46,48]	Frequently used as baseline or comparative classifiers, with performance depending on the dataset, validation strategy, and tuning procedure.
Tree-based and ensemble methods	Decision trees, CART, C4.5, Random Forest, XGBoost, AdaBoost, LightGBM, Bagging	18	[7,9,12,19,23,31,32,33,34,35,36,38,40,41,42,45,46,47]	Most frequently reported family; often among the best-performing models in comparative studies.
Neural networks and deep learning	ANN, MLP, DNN, deep learning models	9	[7,8,12,19,20,33,35,44,46]	Used to model nonlinear patterns, usually in classification settings, although interpretability remains more limited.
Survival and time-to-event models	Cox proportional hazards model	1	[38]	Used to incorporate temporal information in student academic trajectories.
Unsupervised or clustering-assisted methods	K-Means, Gaussian finite mixture models	2	[23,37]	Used to identify student subgroups or trajectories before applying predictive models.
Other computational approaches	Quantized Embeddings Reduction (QED)	1	[32]	Used in a specific high-dimensional representation setting linked to predictive modeling.

**Table 8:** Predictor categories used in graduation-related prediction models.

Predictor Category	Representative Variables	Studies	Included Studies	Evidence of Relevance	Method Used to Establish Relevance
Academic performance	Cumulative GPA, semester GPA, credits earned, failed courses, study duration	23	[7,8,9,12,19,20,23,31,32,33,34,35,36,37,38,39,40,41,43,45,46,47,48]	Frequently reported among the most relevant predictors, especially GPA-related variables, accumulated credits, and academic progression indicators.	Feature importance, SHAP, permutation feature importance, correlation analysis, decision rules.
Sociodemographic variables	Age, gender, ethnicity/race, nationality, domicile, marital status	15	[7,9,12,19,31,32,33,36,38,40,42,43,46,47,48]	Commonly used to contextualize student profiles; age, gender, and underrepresented minority status were reported as relevant in some studies.	SHAP, LIME, permutation feature importance, correlation analysis, statistical significance.
Pre-college or admission variables	High school GPA, entrance exam scores, high school type or accreditation	15	[7,9,12,19,23,31,32,33,38,39,40,42,46,47,48]	Used as early indicators of academic readiness, particularly before university-level performance data become available.	SHAP, correlation analysis, CART goodness of split, *p*-values.
Socioeconomic or financial variables	Parents’ education, family income, scholarships, financial aid, household assets	11	[9,19,31,38,40,42,43,44,45,46,48]	Reported as contextual factors associated with retention, completion, and differential student risk profiles.	Correlation analysis, Cox model coefficients, SHAP, ReliefF.
Institutional variables	Graduation modality, advisor, campus, class type, department	9	[7,12,19,32,34,35,36,40,42]	Used to capture institutional and administrative conditions that may affect progression and completion timing.	Mean decrease in impurity, SHAP, information gain.
Curricular or program-level variables	Major, specialization, study plan hours, course difficulty, specific course grades	9	[7,9,20,23,34,35,37,41,46]	Used to represent program structure, curricular difficulty, and academic progression requirements.	PCA, correlation analysis, permutation feature importance.
Behavioral variables	Class attendance, study time, extracurricular activities, application timing, family influence scale	6	[19,23,33,36,44,45]	Provided additional information on engagement, readiness, and student behavior, although less frequently used than academic predictors.	Correlation heatmap, feature filtering, decision trees.
Longitudinal academic trajectory variables	Years in degree, time since first enrollment, temporal gap	2	[34,35]	Relevant for modeling complex academic trajectories, transfer pathways, and time-to-degree estimation.	Permutation feature importance, RMSE increase.

**Table 9:** Pipeline components reported in graduation-related prediction studies.

Pipeline Component	Studies	Included Studies	Methods Reported
Feature selection	20	[7,9,12,19,23,31,32,33,34,35,36,37,38,40,42,43,44,45,47,48]	Filter methods such as correlation analysis, ReliefF, and Gain Ratio; embedded methods such as mean decrease in impurity and permutation feature importance; wrapper-based selection such as BorutaShap; and statistical selection procedures.
Dimensionality reduction	3	[23,32,37]	Clustering-based reduction, Principal Component Analysis, and Quantized Embeddings Reduction.
Hyperparameter optimization	14	[7,8,9,12,20,31,32,34,35,36,41,43,45,46]	Grid search, GridSearchCV, random search, Optuna, manual tuning, and Particle Swarm Optimization.
Explainability and interpretability	17	[7,9,12,19,23,31,32,33,34,35,36,38,40,42,43,44,47]	SHAP, LIME, permutation feature importance, mean decrease in impurity, decision rules, coefficients, statistical significance, and model-based feature importance.
Bio-inspired optimization actively implemented	2	[19,20]	Particle Swarm Optimization for hyperparameter optimization; Genetic Algorithms and Ant Colony Optimization for feature selection.
Multi-objective optimization	0	None identified	Within the reviewed corpus of 25 studies, no study explicitly formulated graduation prediction as a multi-objective optimization problem. This observation refers only to the analyzed evidence base and not to the entire field of educational data mining or higher education analytics.

**Table 10:** Study-level methodological quality assessment matrix.

Study ID	Q1	Q2	Q3	Q4	Q5	Q6	Q7	Q8	Q9	Q10
[7]	S	S	S	S	S	S	S	S	S	S
[8]	S	S	S	S	S	S	S	S	S	PS
[9]	S	S	S	S	S	S	S	S	S	S
[12]	S	S	S	S	S	S	S	S	S	S
[19]	S	S	S	S	S	S	S	S	S	S
[20]	S	S	S	S	S	NR	S	S	S	NR
[31]	S	S	S	S	S	NR	S	S	S	PS
[23]	S	S	S	S	S	NA	S	S	S	S
[32]	S	S	S	S	S	S	S	S	S	S
[33]	S	S	S	S	S	S	S	S	S	S
[34]	S	S	S	S	S	S	S	S	S	S
[35]	S	S	S	S	S	NA	S	S	S	S
[36]	S	S	S	S	S	S	S	S	S	S
[37]	S	S	S	S	S	NA	S	S	S	S
[38]	S	S	S	S	S	NS	S	S	S	S
[39]	S	S	S	S	NS	NR	S	S	NR	NR
[40]	S	S	S	S	S	NR	S	NS	S	NR
[41]	S	S	S	S	S	S	S	S	S	PS
[42]	S	S	S	S	S	NR	S	S	S	PS
[43]	S	S	S	S	S	S	S	S	S	S
[44]	S	S	S	S	S	S	S	S	S	PS
[45]	S	S	S	S	S	S	S	S	S	NR
[46]	S	S	S	S	S	S	S	S	S	S
[47]	S	S	S	PS	NS	NR	S	S	S	NR
[48]	S	S	S	S	S	S	S	NS	S	NR

Note. S = satisfied; PS = partially satisfied; NS = not satisfied; NR = not reported; NA = not applicable. Q1 = prediction target clearly defined; Q2 = dataset and population described; Q3 = predictors or input features reported; Q4 = preprocessing described; Q5 = validation strategy appropriate; Q6 = class imbalance addressed; Q7 = evaluation metrics reported; Q8 = baseline or model comparison included; Q9 = feature selection, dimensionality reduction, explainability, or optimization reported; Q10 = limitations, generalizability, or bias discussed.

**Table 11:** Descriptive synthesis of evaluation metrics reported in the included studies.

Task Formulation	Metric Family	No. of Studies	Reported Metrics	Representative Studies
Binary or multiclass classification	Standard classification metrics	23	Accuracy, precision, recall, F1-score, confusion matrix, sensitivity, specificity	[19,31,41,46]
Regression, time-to-degree, or time-to-event prediction	Error, explained-variance, and survival metrics	5	RMSE, MAE, MAPE, $R^{2}$, MSE, C-index, IBS	[9,23,35,37,38]
Classification, survival, or risk prediction	Discrimination-oriented metrics	9	AUC, ROC AUC, C-statistic	[12,19,31,32,36,46]
Imbalanced or multiclass classification	Imbalance-aware, chance-corrected, or complementary metrics	4	MCC, kappa, G-Mean	[7,19,32,33]
Classification with restricted reporting	Limited metric reporting	2	Precision and accuracy; APER and accuracy	[42,47]

Note. Categories are not mutually exclusive because some studies reported metrics from more than one task formulation or metric family. Counts therefore refer to the number of studies in each category and should not be summed.

**Table 12:** Methodological gaps and future research directions identified in the reviewed corpus.

Methodological Gap	Evidence from Corpus	Included Studies	Implication	Future Research Direction
Limited adoption of bio-inspired and multi-objective optimization	Only two studies actively implemented bio-inspired optimization within their pipelines: one for hyperparameter tuning and another for feature selection. Within the reviewed corpus, no study explicitly formulated graduation prediction as a multi-objective optimization problem.	[19,20]	The potential contribution of metaheuristics remains insufficiently assessed for feature selection, hyperparameter tuning, model parsimony, and trade-off analysis in educational prediction.	Evaluate lightweight, hybrid, and multi-objective metaheuristic algorithms tailored to educational analytics, including PSO, Genetic Algorithms, Ant Colony Optimization, Differential Evolution, and related bio-inspired approaches.
Limited adoption of local explainability methods	Several studies reported global feature importance, but fewer applied local or model-agnostic interpretability methods such as SHAP or LIME to explain individual predictions.	[7,9,12]	Predictive outputs may be difficult to translate into individualized academic advising or targeted student support.	Expand the use of local XAI methods and integrate them with feature selection strategies to support transparent, personalized, and actionable intervention design.
Limited assessment of algorithmic fairness and bias	Few studies examined model calibration, disparate performance, or fairness across sociodemographic groups.	[9,36]	Predictive models run the risk of inadvertently reflecting historical disparities if subgroup performance is not evaluated.	Incorporate fairness-aware metrics, subgroup validation, and bias mitigation procedures; future bio-inspired pipelines could optimize predictive performance and fairness simultaneously.
Limited evidence of institutional deployment	Most models were evaluated offline using historical datasets, with limited evidence of live deployment in academic information systems or early warning systems.	[12,41]	The practical effect of predictive models on advising decisions, student behavior, and graduation outcomes remains uncertain.	Conduct longitudinal, implementation-oriented, and intervention-based studies in real institutional settings, assessing not only accuracy but also usability, actionability, and decision-support value.
Limited multi-institutional external validation	Most models were developed and tested using data from a single institution, limiting geographic, institutional, and curricular variability.	[36]	Findings on model performance and predictor relevance may be context-dependent.	Validate models across multi-institutional datasets and heterogeneous higher education systems to assess robustness, transferability, and context-sensitive model behavior.
Underuse of dynamic and longitudinal modeling	Many studies relied on static snapshots of student records, while fewer incorporated survival analysis, time-dependent variables, or temporally updated risk indicators.	[34,35,38]	Static models may not capture changes in academic progression, engagement, or risk over time.	Integrate time-to-event models, longitudinal predictors, and dynamic early warning systems that update risk estimates as new data become available.

## Data Availability

The original contributions presented in this study are included in the article/Supplementary Materials. Further inquiries can be directed to the corresponding author(s).

## Supplementary Materials

The following supporting information can be downloaded at: https://www.mdpi.com/article/10.3390/biomimetics11070512/s1, File S1: Screening records, eligibility decisions, and exclusion reasons for the studies identified during the systematic review.
